# DAB2IP attenuates chemoresistance of triple‐negative breast cancer through sequestration of RAC1 to prevent β‐catenin nuclear accumulation

**DOI:** 10.1002/ctm2.1133

**Published:** 2022-12-19

**Authors:** Zhenchong Xiong, Lin Yang, Ning Li, Jianchang Fu, Peng Liu, Peng Sun, Weidong Wei, Xiaoming Xie

**Affiliations:** ^1^ Department of Breast Oncology Sun Yat‐sen University Cancer Center State Key Laboratory of Oncology in South China Collaborative Innovation Center of Cancer Medicine Guangzhou China; ^2^ Department of Pathology Sun Yat‐sen University Cancer Center State Key Laboratory of Oncology in South China Collaborative Innovation Center of Cancer Medicine Guangzhou China; ^3^ Department of Radiation Oncology Nanfang Hospital Southern Medical University Guangzhou China

**Keywords:** DAB2IP, DNA methylation, RAC1, triple‐negative breast cancer, β‐catenin

## Abstract

**Background:**

Although chemotherapy, the most widely used systemic treatment in triple‐negative breast cancer (TNBC), markedly improved the patients’ outcome, chemoresistance always occurs. This study purposed to explore new therapeutic strategies for the treatment of chemoresistance.

**Methods and results:**

The expression and prognostic value of DAB2IP were investigated in TNBC tissues and cell lines. Low DAB2IP expression predicted high mortality risk in TNBC. Inhibition of DAB2IP expression conferred cancer stem cell capacity and chemoresistance in TNBC cell lines. Using murine breast cancer (BC) xenograft models, we evaluated the association with DAB2IP and chemoresistance. DAB2IP inhibited TNBC tumourigenesis and chemoresistance in vivo. Further, we revealed that DAB2IP inhibited β‐catenin nuclear transport through competitive interaction with RAC1 and decreased β‐catenin accumulation in the cell nucleus. Finally, we found that the DNA methylation level was negatively associated with DAB2IP expression in TNBC. Inhibition of DNA methylation restored the DAB2IP expression and attenuated chemoresistance in TNBC.

**Conclusions:**

We revealed that DAB2IP attenuates chemoresistance of TNBC via inhibition of RAC1‐mediated β‐catenin nuclear accumulation. Decitabine treatment results in re‐expression of DAB2IP by inhibiting DNA methylation and could be a potential therapeutic strategy for chemoresistance in TNBC.

## Graphical abstract

DAB2IP attenuates CSC capacity and chemoresistance in TNBC. DAB2IP inhibits β‐catenin nuclear transport through competitive binding to RAC1. Hypermethylation at DAB2IP is associated with low DAB2IP expression and poor prognosis in TNBC. Decitabine treatment inhibits DNA methylation at DAB2IP and improves response to chemotherapy in TNBC.

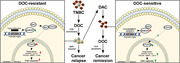

## 1 BACKGROUND

Triple‐negative breast cancer (TNBC), accounting for 15%–20% of breast cancer (BC) population, lacks hormone receptors (estrogen receptor [ER] and progesterone receptor [PR]) expression and Her2 amplification/overexpression.^1^ Since TNBC is insensitive to anti‐Her2 therapy and endocrine‐therapy, chemotherapy becomes the standard of care for TNBC systemic treatment.^2^ Although chemotherapy consisting of paclitaxel and anthracycline has markedly improved the cancer survival in TNBC, many patients develop chemoresistance leading to cancer relapse within 3–5 years.^3^ Finding valuable treatments for chemoresistance has become a critical unsolved medical need.

Development of chemoresistance involves a series of biological processes, such as the drug efflux caused by ‘drug pump’ (ABC transporter) on cell membrane, the abnormal drug metabolism in cancer cells, the dysregulation of cell‐cycle and DNA damage response, the hyper‐activation of pro‐survival signaling and anti‐apoptosis pathways, and the cancer stem cells (CSCs) enrichment..^4^, ^5^, ^6^ CSCs are cell clusters with potential of self‐renewal, intrinsic chemoresistance and multidirectional differentiation.^7^ A recently study has revealed a close association with TNBC and CSC‐related gene signature (epithelial‐mesenchymal transition [EMT]).^8^ Accumulating data indicated that CSCs, enriched in the tumour residual of patients with chemotherapy, results in chemoresistance development in TNBC.^9^, ^10^ Therefore, targeting CSCs is of great value for chemoresistance treatment in TNBC.

Wnt/β‐catenin pathway, abnormally activated in BC, is essential for cancer stemness.^11^ When Wnt signal is absent, β‐catenin would be phosphorylated at Ser33/37 by APC/Axin/GSK‐3β complexes and then degraded by β‐TrCP/Skp pathway‐mediated ubiquitination.^12^ In the presence of Wnt ligand, GSK‐3β is substituted from APC/Axin, causing the increased stability of β‐catenin.^13^ Stable β‐catenin is further translocated into the cell nucleus mediated by RAC1, binding lymphoid enhancer binding factor/T‐cell‐specific transcription factor (LEF/TCF) transcription factors and activating downstream genes (such as Nanog, c‐myc, CD44, and so on).^14^ Besides, RAC1 has been reported to induce β‐catenin S675 phosphorylation through PAK1 resulting in transcriptional active β‐catenin with high stability.^15^ Although RAC1 is a critical activator for Wnt/β‐catenin pathway, the regulatory mechanism underlying RAC1‐mediated β‐catenin nuclear transport in TNBC is unclear and requires further studies.

DAB2IP acts as a tumour‐suppressor commonly loss in several cancers.^16^ DAB2IP prohibits cancer cell proliferation, survival and migration via regulating various oncogenic signalling (including the PI3K‐AKT, RAS‐MAPK, JAK‐STAT pathways).^16^ Accumulating evidence indicated that DAB2IP involved in regulation of CSC capacity. In glioblastoma multiforme (GBM), DAB2IP negatively regulated ATG9B expression and inhibited tumour autophagy by blocking the Wnt/β‐catenin pathway.^17^ In prostate, ovarian and colorectal cancer, DAB2IP expression inhibited CSC‐like phenotypes.^18^, ^19^, ^20^ In BC, DAB2IP inhibited epithelial‐to‐mesenchymal transition (EMT), a critical regulator of the CSC phenotype,^21^ while DAB2IP modulates CSC capacity, and chemoresistance has not been examined in BC. Also, Conway et al. discovered that DAB2IP was more highly methylated in TNBC than in other subtypes of BC.^22^ Whether highly DNA methylation of DAB2IP results in loss of DAB2IP expression and confers CSC capacity in TNBC requires further studies. Recently, we discovered that the low expression of DAB2IP predicted poor prognosis in TNBC. Inhibition of DAB2IP conferred CSC capacity and chemoresistance in TNBC. Further, we discovered that DAB2IP inhibited β‐catenin nuclear accumulation through competitive interaction with RAC1 and deactivated Wnt/β‐catenin pathway. Finally, we discovered that DNA hypermethylation associated with DAB2IP low expression in TNBC. Inhibition of DAB2IP DNA methylation restored its expression and subsequently attenuated chemoresistance in TNBC. Collectively, our study revealed a DAB2IP/RAC1/β‐catenin regulator mechanism that modulates CSC capacity and chemoresistance in TNBC.

## 2 METHODS

### 2.1 Patients and tissue specimens

Paired BC and tumour‐adjacent noncancerous tissue obtained by operational resection was used for detection of DAB2IP expression (mRNA and protein). The tumour‐adjacent non‐cancerous tissue is defined as noncancerous breast tissues located more than 3 cm from the tumour boundary. Formalin‐fixed paraffin‐embedded (FFPE) tissues of TNBC (*n* = 304) at Sun Yat‐sen University Cancer Center (from 2000 to 2015) were collected for DAB2IP protein expression and DNA methylation analysis. The diagnosis of TNBC was determined according to the status of hormone receptor (estrogen receptor [ER] and progesterone receptor [PR]) and Her2 receptor. Hormone receptor (HR) (ER and PR) expression of BC tissues is measured by immunohistochemistry (IHC). Nuclei staining of less than 1% of cells is considered ER/PR negative.^23^ Her2 status of BC tissues is determined by either IHC to detect membrane HER2 expression or by FISH to assess gene copy number.^24^ For immunohistochemistry (IHC) assay, HER2 staining was defined as: negative (0/1+), equivocal (2+) and positive (3+). For FISH assay, HER2 status was defined as: negative (HER2/CEP17 ratio < 2.0 and HER2 copy number < 4), equivocal (HER2/CEP17 ratio < 2.0 and HER2 copy number ≥ 4 but < 6) and positive (HER2/CEP17 ratio ≥ 2.0 or HER2 copy number ≥ 6 regardless of ratio).^24^ For tissues with equivocal IHC staining of HER2 receptor, BC tissues were conducted with FISH assay. HER2 (‐) status is defined as: negative staining (IHC) or equivocal staining (IHC) and negative FISH status. HER2 (+) status is defined as: cases with positive staining (IHC) or cases with equivocal staining (IHC) and positive FISH status.

The pathological and clinical features were displayed in Table S1. Patient prognosis data were obtained via a telephone interview or outpatient review. This study was complied with the Helsinki Declaration and was approved by the institutional review board.

### 2.2 Cell culture and transfection

TNBC cell line (MDA‐MB‐231, HCC1806, BT‐549, SUM159PT and MDA‐MB‐468) were maintained as follow: foetal bovine serum (FBS) (HyClone; 10%) and penicillin/streptomycin (1%) were mixed with Dulbecco's modified eagle medium (DMEM) (Invitrogen), RPMI‐1640 (Invitrogen) or formulated Leibovitz's L‐15 Medium (Invitrogen); cells were cultured with CO_2_ (5%) at 37°C.^25^ Luminal A BC cell lines (T47D and MCF‐7 [human breast cancer cell line]), luminal B BC cell lines (ZR‐75‐30 and BT474) and HER2 BC cell lines (SKBR3) were maintained as follow: FBS (HyClone; 10%) and penicillin/streptomycin (1%) were mixed with DMEM (Invitrogen), RPMI‐1640 (Invitrogen), Hybri‐Care Medium (American Type Culture Collection) or McCoy's 5a Medium Modified (Invitrogen); cells were maintained with CO_2_ (5%) at 37°C.^25^ For low dose of decitabine (DAC) (Sigma # 1165204) treatment, cells were plated for 1 day and incubated in daily refreshed medium containing 100 nM of decitabine for 3 days.^26^, ^27^ For docetaxel (DOC) treatment, cells were plated for 1 day and cultured with medium containing the indicated dose of docetaxel (DOC). DOC‐resistant TNBC cells (MDA‐MB‐231‐Doc‐R and HCC1806‐Doc‐R) were established in medium containing DOC via a two‐fold increase in concentration (1‐64 nM for MDA‐MB‐231 and 2–128 nM for HCC1806) every 4 weeks. For doxorubicin (DOX) treatment, cells were plated for 1 day and cultured with medium containing the indicated dosages of doxorubicin.

*DAB2IP* cDNA (corresponding to ENST00000259371.6) was amplified and cloned into the pEZ‐Lv201 vector. The psi‐LVRU6GP vectors containing short‐hairpin RNA (shRNA) were used to inhibit the *DAB2IP* and *RAC1* expression. Cells were cultured with lentivirus generated with pEZ‐Lv201‐puro‐DAB2IP‐Flag/vector, psi‐LVRU6GP‐shRNAs/control or pEZ‐Lv201‐puro‐RAC1‐HA for 72 h and selected with puromycin to induce stable cell line.

### 2.3 Quantitative real‐time polymerase chain reaction

For RNA extraction: Fresh BC and tumour‐adjacent tissues were added to lysis buffer and ground; TNBC cells were digested and then added to lysis buffer. Then, RNA extraction from fresh tissues and cells were conducted following the manufacturer's protocol (Takara; cat. # 9767). After RNA extraction, the absorbance and concentration of each sample were detected, and the OD value of RNA should be 1.8‐2. For cDNA synthesis: 500 ng per sample was used (Takara; cat. # RR036Q). The quantitative real‐time polymerase chain reaction (qRT‐PCR) assay was conducted with TB Green Fast qPCR Mix (TaKaRa; cat. # RR430A) following the manufacturer's instructions. The specific primer sequences are showed in Table S2.

*GAPDH* was set as housekeeping gene. Three replicates per sample were conducted in the qRT‐PCR assay. Cycle threshold (Ct) value of each reaction was calculated using Crossing Point method.^28^ The Ct value of each reaction was applied to assess the relative mRNA expression of housekeeping gene or target genes for each sample using the △△Ct method: (1) △Ct value (ΔCt = Ct(target gene) − Ct(reference gene)) is conducted for reference gene calibration. (2) △△Ct value (ΔΔCt = ΔCt(target sample) − ΔCt(reference sample)) represents the expression variation of target gene for each sample comparing to the control group. (3) Relative gene expression of each sample is calculated as 2^‐△△Ct.^29^

### 2.4 Protein lysates and Western blotting

RIPA lysis buffer (Sigma‐Aldrich) was added to Cells for protein extraction. For detection of total β‐catenin level, protein lysis was treated with phosphatase (Beyotime # D7027) for 60 min at 37°C. The bicinchoninic acid (BCA) assay (Pierce, Rockford, USA) was applied to evaluate the protein concentrations. Western blotting was conducted following previously published protocol.^30^ Antibodies used in our studies were listed as follow: anti‐DAB2IP polyclonal rabbit antibody (1:2000 dilution; Abcam # ab87811), anti‐β‐catenin rabbit antibody (1:1000 dilution; CST # 8480), anti‐Phospho‐β‐catenin (Ser 675) rabbit antibody (1:1000 dilution; CST # 4176), anti‐Phospho‐β‐catenin (Ser 33/37) rabbit antibody (1:1000 dilution; CST # 2009), anti‐RAC1 rabbit antibody (1:2000 dilution; Sigma # 05–389), anti‐GAPDH monoclonal mouse antibody (1:1,000 dilution; CST # 51332) and goat anti‐mouse/rabbit IgG secondary antibody (1:2,000 dilution; ThermoFisher # 31430/31460).

### 2.5 IHC

Protein expression of DAB2IP in FFPE tissues was evaluated by IHC as previously described. Anti‐DAB2IP polyclonal rabbit antibody (14 μg/ml; Sigma# SAB4301073) was used. Two pathologists separately assessed the immunostaining intensity. Scores comprised the proportion of cells with positive staining and the staining intensity. We scored the intensity of staining as such: (1) negative, (2) weak (light yellow), (3) moderate (yellow brown) and (4) strong. The percentage of cells with positive staining was stratified as such: 0, no positive cells; (1) <10%, (2) 10%–35%, (3) 35%–75% and (4) > 75%. The staining index (SI) was calculated by multiplying the proportion of positively stained cells with the intensity score of staining, and the SI was applied to assess the protein expression level. SI ≥ 8 and SI < 8 were defined as high expression and low expression, respectively.

### 2.6 Tumour‐sphere culture and isolation of CSC‐like cells

MDA‐MB‐231 with high DAB2IP expression/low DAB2IP DNA methylation and HCC1806 with low DAB2IP expression/high DAB2IP were cultured (1,000 cells/ml) in ultralow attachment dishes (Corning) in DMEM/F‐12 medium with epidermal growth factor (20 ng/ml, Invitrogen), insulin (10 mg/ml, Sigma‐Aldrich), basic fibroblast growth factor (10 ng/ml, Invitrogen) and 1% B27 (Invitrogen, Carlsbad, CA). Specifically, cells are digested and suspended in 1× PBS, and cell suspension was added with Trypan Blue to evaluate the cell viability. Then, TNBC cells were seeded in tumoursphere medium (1000 cells/ml), and spheres were counted after 7 days or collected for extraction of RNA, DNA or protein.^31^ For wnt3a treatment, tumour spheres were stimulated with Wnt3a (250 ng/ml, R&D Systems; 0, 30, 60 or 90 min).

Tumour spheres cultured in ultralow attachment dishes for 7 days were incubated in neutral buffered formalin (10%) overnight and embedded in 4% agar before paraffin embedding. The embedded spheres were cut into slices for subsequent immunocytochemistry (ICC) and immunofluorescence (IF) analyses.

### 2.7 Flow cytometry

Before analysis of cell apoptosis or of the CSC population using a flow cytometer (CytoFLEX; Beckman Kurt), 2 × 10^4^ cells were washed and suspended. For the cell apoptosis assay, the Annexin V/PI staining (Invitrogen) was conducted following the manufacturer's methods. For the analysis of the CSC population, CD44 (Tonbo Biosciences) and CD24 (Invitrogen) were used to identify the CSC population, while propidium iodide (PI, Sigma‐Aldrich) was used to identify live cells.

### 2.8 Cell viability assay

Two thousand Cells were added in plates (96‐well) and treated with decitabine or docetaxel. The cell viability was analyzed via cell counting kit‐8 (CCK‐8) following the manufacturer's protocol.

### 2.9 Limiting dilution assay and xenograft tumour model

BALB/c nude mice (female, 4–5 weeks old, 18–20 g) were obtained from the Beijing Vital 17 River Laboratory Animal Technology Company Limited (China). 1 × 10^6^ luciferase‐tagged cells were subcutaneously inoculated into the mammary fat pads (right) of mice, and then tumour initiation was detected every 4 day since the 8th day after cancer cell inoculation. DAC/DOC therapy started when the tumour volume was about 200 mm^3^. When treated with DAC, mice were intraperitoneally injected with DAC at low dosage (2.5 mg/kg, i.p. four injections per week), which was calculated refer to the dosage for human beings (7.5 mg/m^2^) (the dosage conversion was conducted based on the FDA recommendation: https://www.fda.gov/downloads/drugs/guidances/ucm078932.pdf); the dose of 7.5 mg/m^2^ reaches a plasma concentration as much as 100 nM in human beings.^32^ When treated with DOC treatment, mice were intraperitoneally injected with DOC (15 mg/kg, i.p. per week). When treated with the combination therapy (DAC + DOC), mice were intraperitoneally injected with DAC on D1‐4 and treated with DOC on D5 every week. The tumour burden was assessed through luciferase activity (Caliper) or the tumour volume ([length × width^2^]/2).

The limiting dilution experiment was used to assess the tumour initiation ability in vivo. TNBC cells added with Matrigel (Corning) were subcutaneously inoculated into four mammary fat pads of non‐obese diabetic/severe combined immunodeficient (NOD/SCID) mice (Beijing Vital 17 River Laboratory Animal Technology Company Limited, China). The tumour‐initiation ability was evaluated through extreme limiting dilution analysis and luciferase signal.^33^ This study was approved by the Institutional Animal Care and Use Committee of SYSU.

### 2.10 TdT‐mediated dUTP Nick‐End Labelling assay

The TdT‐mediated dUTP Nick‐End Labelling (TUNEL [terminal deoxynucleotidyl transferase dUTP nick end labeling]) experiment was conducted following the manufacturer's instruction (Genecopoeia; cat. # A050E). The slices of FFPE tissue were deparaffinized, rehydrated and incubated in dilute proteinase K solution and DNase buffer at 25°C for .5 h and 5 min, respectively. The tissue slices were incubated with the TdT reaction mixture at 25°C in the dark. The apoptotic cells and cell nuclei were labelled with Andy Fluor 647‐Streptavidin staining buffer and DAPI and detected by fluorescence microscopy. Note that 500–1000 cells per case were counted, and the proportion of TUNEL‐stained cell was calculated.

### 2.11 ICC and IF

For ICC, cells grown in attached culture or tumour spheres were paraffin‐embedded, and the paraffin blocks were cut into slices. The slices of FFPE tissue have been deparaffinized, rehydrated and heated with citrate buffer (pH = 6.0) to retrieve antigen. After blocking antigens with normal goat serum for 2 h, the slices were added with anti‐DAB2IP polyclonal rabbit antibody (14 μg/ml; Sigma# SAB4301073) at 4°C overnight. Ten per cent Mayer's hematoxylin and the goat anti‐rabbit IgG secondary antibody (1:1000) were used to label DAB2IP and the cell nucleus, respectively. Images were captured through a microscope (Olympus BX51).

For immunofluorescence (IF), slices were deparaffinized, rehydrated, antigen‐retrieved and antigen‐blocked. Then, the antigens were labelled with anti β‐catenin rabbit antibody (1:100 dilution; CST # 8480) at 4°C overnight. A goat anti‐mouse/rabbit IgG secondary antibody (Alexa Fluor 488/594; Invitrogen) was used to label β‐catenin, while DAPI (Sigma‐Aldrich) was used to label the cell nuclei. Images were taken using a high‐resolution laser confocal microscope (Zeiss).

### 2.12 Protein immunoprecipitation, protein competitive assays and Pull‐Down assay

The competitive assay was conducted using protein immunoprecipitation (IP). Specifically, cells mixed with IP lysis buffer were centrifuged to extract cellular protein in the supernatant. The concentration of cellular protein was assessed via the BCA assay kit (ThermoFisher). Cell lysates were incubated with antibodies and Flag affinity agarose (Sigma # A2220), HA affinity agarose (Sigma # A2095), or protein G‐conjugated agarose (Sigma # IP04), overnight at 4°C. Next, the beads with affinity‐bound proteins were eluted by wash buffer (10 mM HEPES, 150 mM NaCl, .1% NP40, pH 7.4), and the bounded proteins were washed out using glycine (1 M, pH 3.0). The eluted protein was used for western blotting using anti‐DAB2IP, anti‐RAC1 and anti‐β‐catenin antibody. RAC1 activity was evaluated by Active Rac1 Pull‐Down and detection kit (ThermoFisher # 16118) following the manufacturer's protocol.

### 2.13 DNA methylation analysis

DNA was obtained from FFPE specimens (thicknesses < .1 cm) (Qiagen Kit; cat. # 1071592) or TNBC cells (Omega, Tissue DNA Kit; cat. D3396‐02) and underwent bisulfite conversion (Qiagen Kit; cat. # 59824) following the manufacturers’ protocol. Primer for Bisulfite converted DNA sequencing PCRs (bisulfite sequencing PCR) was determined based on the MethPrimer.^34^ PCR assay with bisulfite converted Genomic DNA (100–500 ng) was performed following the manufacturer's protocol (Takara EpiTaq HS Kit; cat. # R110Q). The products of PCR cloned into the pGEM‐T Easy Vector System were sequenced with the M13F universal primer. Six clones of each sample were assessed, and the mean methylation degree was calculated. The level of DNA methylation was calculated as follow: number of methylated‐loci/(number of methylated and unmethylated loci). The DAB2IP methylation level was assessed in TNBC FFPE tissues (*n* = 304), and promoter methylation analysis was failed in 78 tissues since low DNA concentration (<100 ng) or tissue DNA degradation. Hypermethylation case is defined as BC tissues, which was methylated at over 50% of CpG sites at promoter region; hypomethylation case is defined as BC tissues, which was methylated at less than 50% of CpG sites at promoter region.

### 2.14 Statistical methods

Statistical analyses were performed via the SPSS software (v20.0). The clinicopathological features of patient groups (low/high expression) were assessed by the χ^2^ test. Survival analysis was conducted using the Kaplan–Meier test and the log‐rank test. The survival data were processed via univariate and multivariate Cox regression model. *p* < .05 was considered statistically significant.

## 3 RESULTS

### 3.1 Low DAB2IP expression associates with poor prognosis in TNBC

**FIGURE 1:**
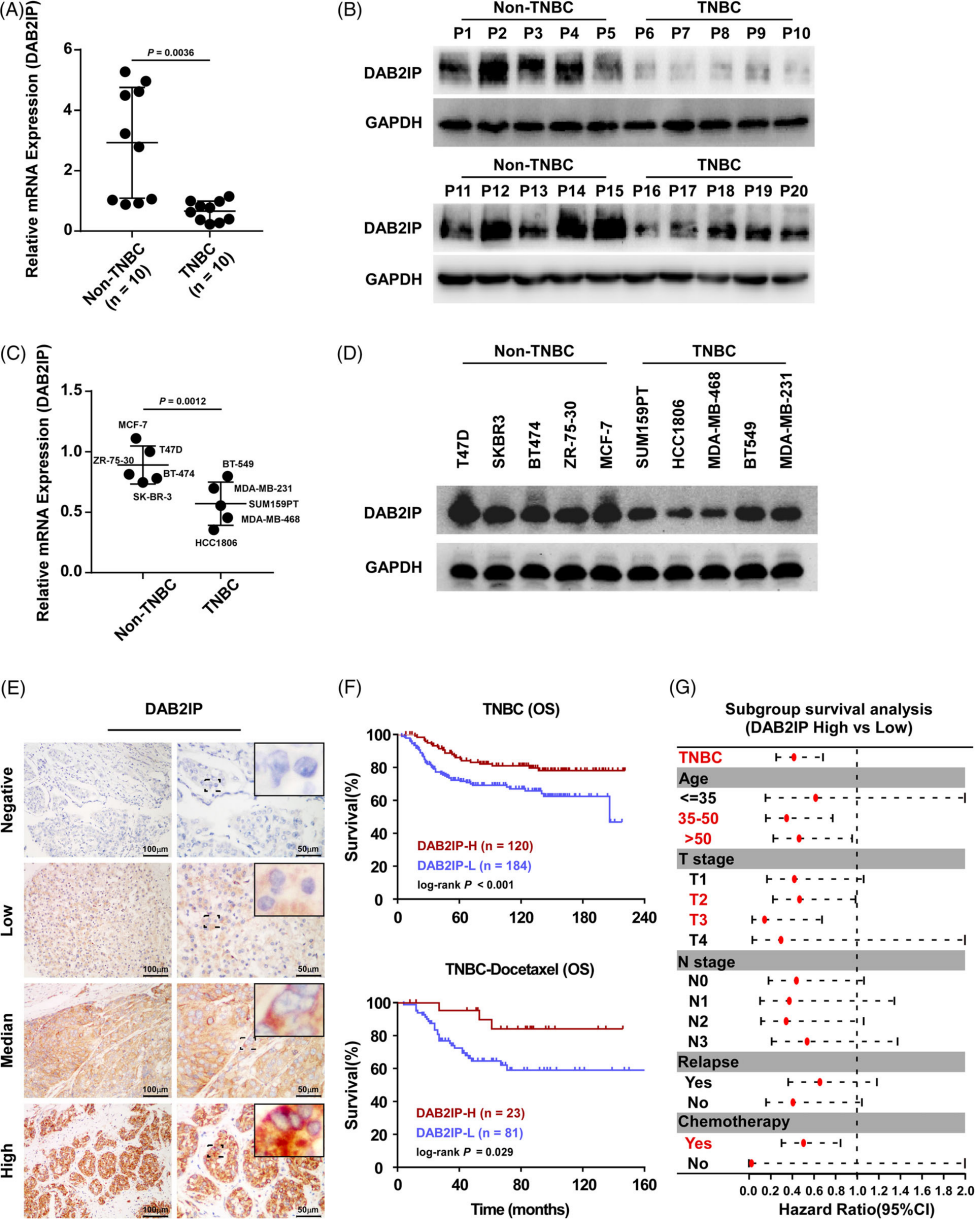
Low DAB2IP expression associates with poor prognosis in triple‐negative breast cancer (TNBC). (A and B) The mRNA of *DAB2IP* (*Transcript 1*) (A) and protein (B) expression levels of DAB2IP in non‐TNBC and TNBC tissues. (C and D) The mRNA of *DAB2IP* (*Transcript 1*) (C) and protein (D) expression levels of DAB2IP in non‐TNBC and TNBC cell lines. (E) Representative immunohistochemistry (IHC) image of DAB2IP expression in TNBC specimens. (F) Kaplan–Meier curve of OS for TNBC patients (upper panel) and TNBC patients with docetaxel‐based chemotherapy (lower panel) using high expression of DAB2IP (DAB2IP‐H) versus low expression of DAB2IP (DAB2IP‐L). (G) Forest plot of the OS subgroup analysis with respect to DAB2IP expression: DAB2IP high versus DAB2IP‐Low. In (A) and (C), three replicates of each sample are conducted, data are presented as the mean ± SD, and *p*‐values were determined by two‐tailed Student's *t* test. In (F), *p*‐values were determined by log‐rank test. In (G), hazard ratio and *p*‐values were determined by univariate Cox regression. OS, overall survival.

To explore the function of DAB2IP in TNBC, DAB2IP expression was detected in 20 paired BC tissues and tumour‐adjacent noncancerous tissues. We detected three transcripts of *DAB2IP* and discovered that only the mRNA expression of *Transcript 1* was decreased in BC tissues compared to tumour‐adjacent noncancerous tissues (Figure S1A–C). Consistently, the mRNA expression of *Transcript 1* was lower than *transcript 2* and *3* in tumour tissues (Figure S1D). IHC assay showed that tumour tissues exhibited more intense DAB2IP staining comparing to tumour adjacent noncancerous tissues (Figure S1E). By Western blotting, we discovered that the expression of DAB2IP was significantly decreased in TNBC tissues comparing to non‐TNBC tissues (Figure 1A,B). The protein and mRNA level of DAB2IP were lower in TNBC cells (Figure 1C,D).

To investigate the prognostic value of DAB2IP in TNBC, we detected DAB2IP expression in 304 paraffin‐embedded TNBC specimens. DAB2IP staining was detected in the cytoplasm of TNBC cells and in some stromal cells as yellow‒brown granules, while DAB2IP staining was observed only in infiltrating lymphocytes (Figure 1E). Pathological and clinical features of patients are displayed in Table S1. Survival analysis indicated that low DAB2IP expression predicted poor outcomes in TNBC patients (Figure 1F). In patients accepted chemotherapy (including DOC‐based and non‐DOC‐based regimen), high DAB2IP expression associated with better survival (Figure 1F and Figure S1F). Subgroup analysis indicated that low DAB2IP expression predicted high mortality risk in TNBC patients ages over 35, T2‐3 stage and chemotherapy (Figure 1G).

### 3.2 DAB2IP inhibition confers CSC capacity and chemoresistance in TNBC cells

**FIGURE 2:**
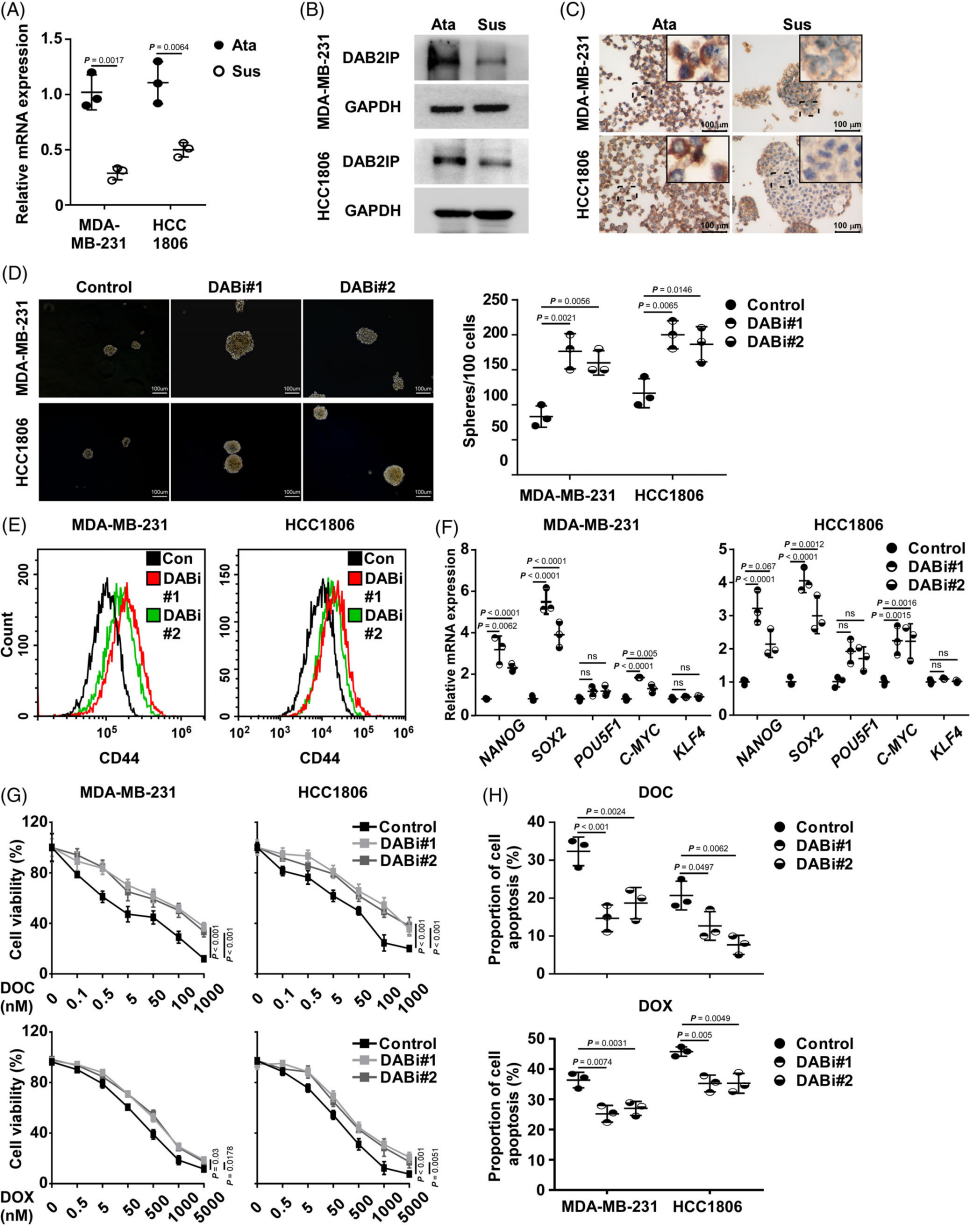
DAB2IP inhibition confers cancer stem cell (CSC) capacity and chemoresistance in triple‐negative breast cancer (TNBC) cells. (A) Comparison of the mRNA expression of *DAB2IP* (*Transcript 1*) between attachment‐cultured cells and suspension‐cultured cells. *GAPDH* was used as an internal control. (B) The protein expression of DAB2IP in attachment‐cultured or suspension‐cultured cells. GAPDH was used as an internal control. (C) Representative images of DAB2IP staining using immunocytochemistry (ICC). Scale bar: 100 μm. (D) Tumour sphere formed by control and DAB2IP‐silencing (DABi#1 and DABi#2) TNBC cells: (left panel) representative images of tumour spheroids; (right panel) quantification of tumour spheroids. (E) Flow cytometry for CD44 cell surface expression in control and DAB2IP‐silencing (DABi#1 and DABi#2) TNBC cells. (F) Quantitative real‐time polymerase chain reaction (qRT‐PCR) analysis of *NANOG*, *SOX2*, *POU5F1* and *C‐MYC* in control and DAB2IP‐silencing (DABi#1 and DABi#2) cells. (G) Cell viability was assessed in control and DAB2IP‐silencing (DABi#1 and DABi#2) cells treated with docetaxel (DOC). (H) Cell apoptosis was evaluated by an Annexin V/PI assay using flow cytometry. Control and DAB2IP‐silencing (DABi#1 and DABi#2) cells were treated with DOC (5 nM, 48 h; upper panel) and DOX (.1 μM, 48 h; lower panel). In (A), three replicates of each reaction are conducted, and data are presented as the mean ± SD, and *p*‐values were determined by two‐tailed Student's *t* test. In (D), (F) and (H), three replicates of each reaction are conducted and data are presented as the mean ± SD, *P*‐values were determined by one‐way ANOVA. In (G), three replicates of each reaction are conducted, data are presented as the mean ± SD, and *p*‐values were determined by two‐way ANOVA. ANOVA, analysis of variance.

A previous study indicated that DAB2IP is a cancer suppressor; and loss of DAB2IP triggers EMT and distant metastasis in BC.^21^ As Lu et al. reported that cancer cells that underwent EMT exhibited enhanced CSC capacity,^35^ we further explored whether DAB2IP inhibition confers CSC capacity in TNBC. Self‐renewal capacity is the hallmark for CSCs and contributes to the capacity to form tumour spheres. Accordingly, Ponti et al. reported that BC cells in suspension culture exhibited CSC‐related features (including positive staining for CD44 and negative staining for CD24, tumour‐initiating capacity in vivo).^1^ Thus, BC cells with CSC properties can be propagated in vitro as mammospheres under suspension culture.^36^ We enriched TNBC cells with CSC capacity through suspension culture and assessed DAB2IP expression. The mRNA and protein level of DAB2IP was down‐regulated in suspension‐cultured spheres comparing to attachment‐cultured cells (Figure 2A,B). No significant changes of the *Transcript 2* and *Transcript 3* mRNA expression were observed in suspension‐cultured TNBC cells (Figure S2A). ICC showed that attachment‐cultured cells exhibit higher DAB2IP expression than suspension‐cultured spheroids (Figure 2C and Figure S2B). Next, we established DAB2IP‐overexpression/inhibition stable cell lines in two TNBC cell lines (Figure S2C,D). Tumour‐sphere assay indicated that DAB2IP‐inhibition enhanced and DAB2IP‐overexpression attenuated self‐renewal ability in TNBC cells (Figure 2D and Figure S2E). CD44^hi^ population enrichment was induced in cells with DAB2IP‐inhibition and prohibited in cells with DAB2IP‐overexpression (Figure 2E and Figure S2F). Moreover, several self‐renewal regulators (such as *SOX2* and *NANOG*) were up‐regulated in cells with DAB2IP‐inhibition but down‐regulated in cells with DAB2IP‐overexpression (Figure 2F and Figure S2G).

As DAB2IP inhibition conferred enrichment of CSC, we explored whether DAB2IP inhibition confers chemoresistance in TNBC. Under normal condition, DAB2IP‐inhibition increased TNBC cell viability while DAB2IP‐overexpression decreased cell viability (Figure S2H). When treated with chemotherapy, mRNA expression of DAB2IP was significantly decreased (Figure S2I). Cells with DAB2IP‐inhibition displayed higher viability than control cells under DOC treatment (Figure 2G). With flow cytometry, our data discovered a lower percentage of cell apoptosis in DAB2IP‐silenced cells than in control cells (Figure 2H). Consistently, DAB2IP‐overexpression induced the lethal effect of DOC on TNBC cells (Figure S2J,K). To verify the influence of DAB2IP on chemoresistance, we treated TNBC cells with doxorubicin (DOX), another widely used chemotherapeutic agent for TNBC. With DOX treatment, DAB2IP‐inhibition significantly increased cell viability (Figure 2G,H) and DAB2IP‐overexpression decreased cell viability in TNBC cells (Figure S2J,K). Collectively, we discovered that DAB2IP inhibition confers CSC capacity and chemoresistance in TNBC cells.

### 3.3 DAB2IP inhibition induces tumourigenesis and chemoresistance in TNBC

**FIGURE 3:**
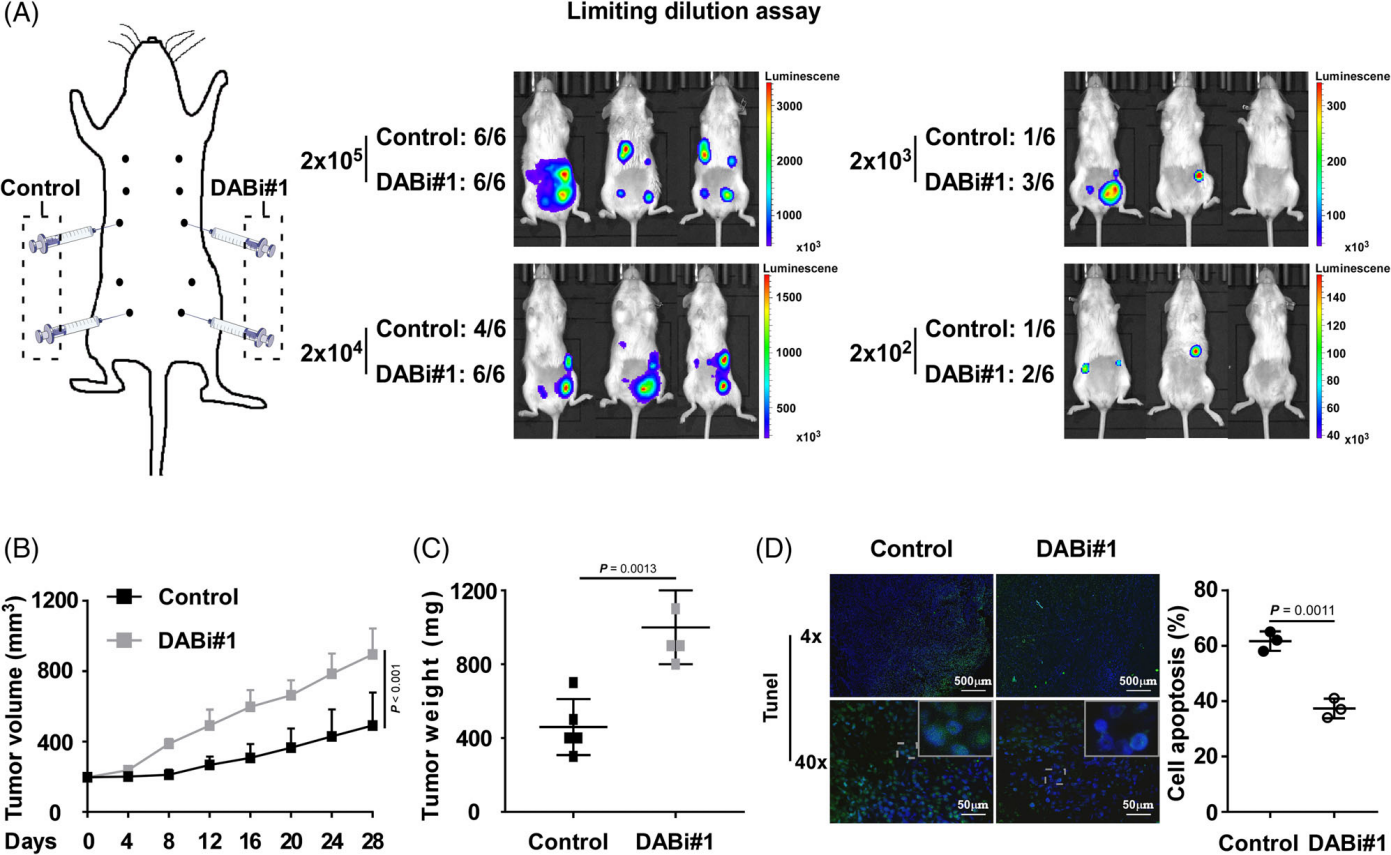
DAB2IP inhibition induces tumourigenesis and chemoresistance in triple‐negative breast cancer (TNBC). (A) The limiting dilution assay was used to evaluate the tumour initiation capacity. The indicated number of MDA‐MB‐231‐control cells or MDA‐MB‐231‐DABi#1 (with DAB2IP inhibition) cells was mixed with Matrigel and subcutaneously injected into right (MDA‐MB‐231‐control) and left (MDA‐MB‐231‐DABi#1) mammary fat pads of female NOD/SCID mice. A schematic diagram of the limiting dilution assay is shown (left panel). The tumour initiation frequency was assessed by luciferase activity (middle and right panel). (B) Volumes of tumours in control and DAB2IP‐silencing (DABi#1) groups (*n* = 5/group). Mice were treated with docetaxel (DOC) (15 mg/kg, i.p. weekly) when tumours reached a volume of 200 mm^3^. Tumour volumes were measured every 4 days and are shown by tumour growth curves. (C) Tumour weights of tumours in control and DAB2IP‐silencing (DABi#1) groups (*n* = 5/group). (D) Representative images (left panel) and quantification (right panel) of TUNEL staining of apoptotic cells in control and DAB2IP‐silencing (DABi#1) tumours treated with DOC (*n* = 5/group). Cell nuclei were labelled with DAPI (in blue), and the apoptotic cell fraction was labelled with TUNEL (in green). Scale bars: 50 and 500 μm. In (B), three replicates of each reaction are conducted, data are presented as the mean ± SD, and *p*‐values were determined by two‐way ANOVA. In (C) and (D), data are presented as the mean ± SD, and *p*‐values were determined by two‐tailed Student's *t* test. ANOVA, analysis of variance.

As CSC resistance to chemotherapy results in tumour initiation and cancer relapse, we conducted the limiting dilution assay to investigate the influence of DAB2IP on the tumourigenesis of TNBC. Four mammary fat pads of each NOD/SCID female mouse were inoculated with TNBC cells with DAB2IP‐overexpression/inhibition or control cells at concentrations ranging from 2 × 10^3^ to 2 × 10^5^ cells (Figure 3A and Figure S3A). All injections of cells with silenced DAB2IP induced tumour initiation at a concentration of 2 × 10^4^, while two of six injections of control cells failed to initiate tumours. At a concentration of 2 × 10^3^–10^2^, a lower incidence of tumour initiation was showed in control cells than in cells with DAB2IP‐inhibition (Figure 3A). Conversely, our data showed an increased frequency of tumour initiation in control cells comparing to the frequency in cells with DAB2IP‐overexpression (Figure S3A). Thus, DAB2IP attenuated the ability of tumourigenesis in TNBC (control vs. DABi#1, *p* = .002; vector vs. DAB2IP, *p* < .005; Table 1).

**TABLE 1:** Incidence of tumours in NOD/SCID mice

	**Number of outgrowths**				
**Number of cells**	**Vector**	**DAB2IP**		**Control**	**DABi#1**
2 × 10^5^	6/6	5/6		6/6	6/6
2 × 10^4^	5/6	2/6		4/6	6/6
2 × 10^3^	1/6	0/6		1/6	3/6
2 × 10^2^	1/6	0/6		1/6	2/6

***p*‐Value**	**Stem cell frequency**		***p*‐Value**	**Stem cell frequency**	
<.005	1/9353	1/92,028	.002	1/13,788	1/1895

In animal models, we validated the DAB2IP protein expression in tumours formed by DAB2IP‐inhibition/overexpression TNBC cells using Western blotting and IHC assay (Figure S3C). With DOC treatment, DAB2IP inhibition associated with faster growth rate and bigger volume of tumour while DAB2IP overexpression associated with slower growth rate and smaller tumour volume (Figure 3B,C and Figure S3D,E). Also, our data showed that DAB2IP inhibition significantly associated with decreased percentage of apoptotic cell and overexpression of DAB2IP associated with increased percentage of apoptotic cell in tumours with DOC treatment (Figure 3D and Figure S3F).

### 3.4 DAB2IP inhibition activates Wnt/β‐catenin signalling

**FIGURE 4:**
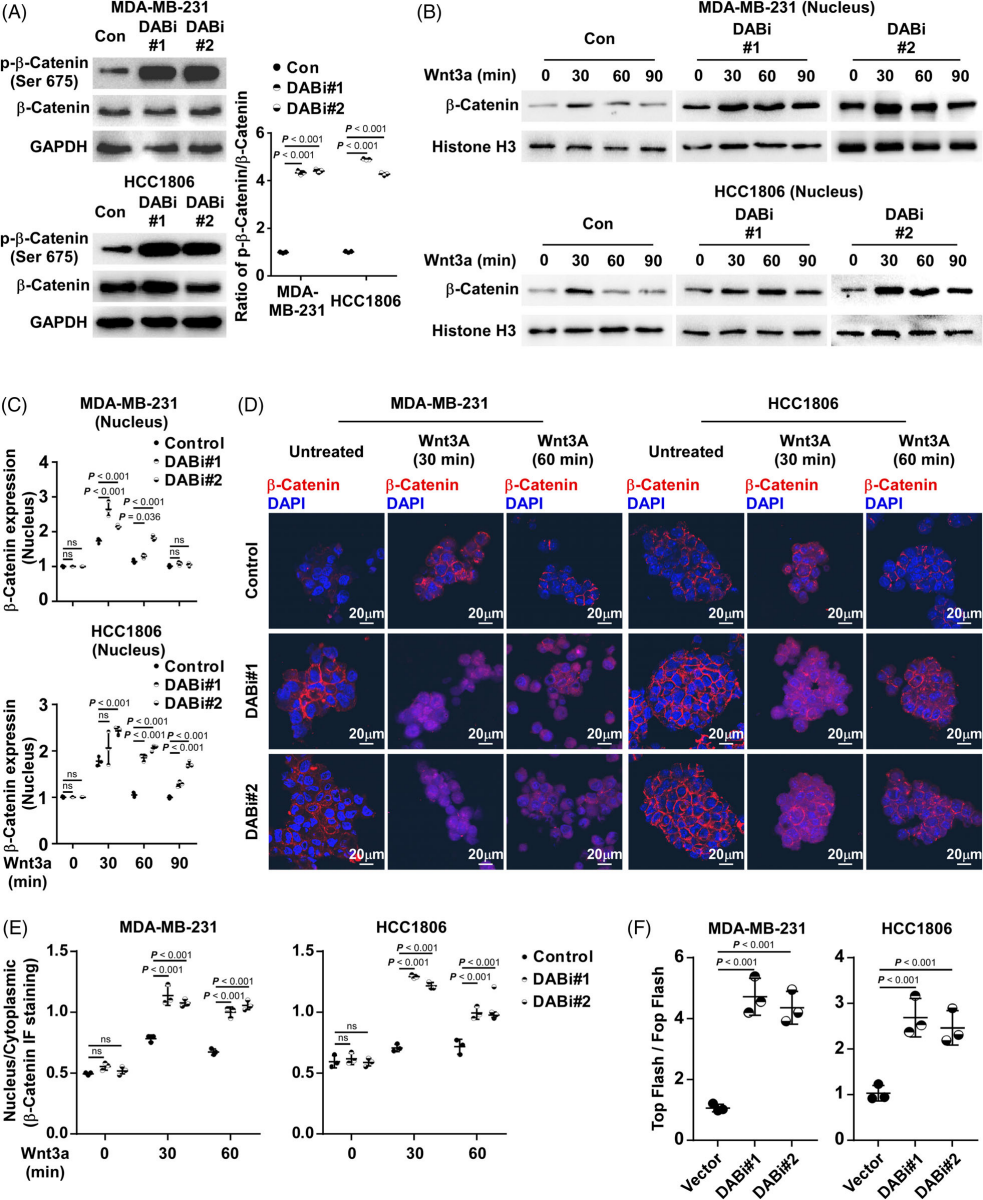
DAB2IP inhibition activates Wnt/β‐catenin signalling. (A) Western blotting (left panel) of phospho‐β‐catenin (Ser 675) and β‐catenin and quantification (right panel) of phospho‐β‐catenin (Ser 675)/β‐catenin ratio in control and DAB2IP‐silencing (DABi#1 and DABi#2) cells with Wnt3a stimulation (30 min). GAPDH was used as an internal control. (B and C) Western blotting (B) and quantification (C) of nucleus β‐catenin in control and DAB2IP‐silencing (DABi#1 and DABi#2) triple‐negative breast cancer (TNBC) cells stimulated with Wnt3a. Histone H3 was used as an internal control. (D and E) Representative images (D) and quantification (E) of β‐catenin (in red) staining in tumour spheroids formed by control and DAB2IP‐silencing (DABi#1 and DABi#2) cells. Cell nuclei were labelled with DAPI (in blue). Images were captured using confocal microscopy. Scale bar: 20 μm. (F) Luciferase reporter assays of TOP Flash/Fop Flash reporters. In (A), (C), (E) and (F), three replicates of each reaction are conducted, data are presented as the mean ± SD, and *p*‐values were determined by one‐way ANOVA. ANOVA, analysis of variance.

β‐catenin, downstream of Wnt signalling, is critical for CSC ability in BC. To investigate the mechanism of CSC enrichment inhibition by DAB2IP, we investigated the association between Wnt/β‐catenin pathway activation and DAB2IP expression. DAB2IP did not alter either the expression of β‐catenin or the expression of phospho‐β‐catenin (Ser33/37) (Figure S4A). However, we discovered that DAB2IP inhibition associated with the increased expression of phospho‐β‐catenin (Ser675) in TNBC cells (Figure 4A). A previous study has reported that phosphorylation at Ser675 promotes nuclear accumulation and increases the β‐catenin transcriptional activity.^37^ To investigate whether inhibition of DAB2IP conferred the nucleus expression of β‐catenin, we treated TNBC spheroids using Wnt3a (250 ng/ml), the stimulator for β‐catenin pathway. Through nuclear protein extraction, we observed that inhibition of DAB2IP increased the nucleus expression of β‐catenin in TNBC cells stimulated with Wnt3a (Figure 4B,C). Through IF, we observed more intense nuclear β‐catenin staining in DAB2IP‐inhibiting cells than that of control cells (Figure 4D,E). As β‐catenin regulates downstream gene expression in cooperation with LEF/TCF transcription factors, we used a TCF reporter plasmid/TCF reporter plasmid containing mutated TCF binding sites (TOP/FOP) dual luciferase reporter system to assess β‐catenin transcriptional activity. With the binding sequences of LEF/TCF transcription factors inserted into the dual luciferase plasmid, the dual luciferase reporter system revealed β‐catenin transcriptional activity through the regulation of luciferase expression. We observed that DAB2IP‐inhibition was associated with increased β‐catenin transcriptional activity (Figure 4F). Consistently, TNBC cells with DAB2IP‐overexpression exhibited decreased Wnt/β‐catenin signalling activity compared to control cells (Figure S4B–G). Further, DAB2IP‐inhibition associated with increased expression of β‐catenin downstream genes (CD44, PROM1 and MMP7) in TNBC cells, while DAB2IP‐overexpression associated with decreased expression of CD44, PROM1 and MMP7 (Figure S4H). These data indicated that DAB2IP modulates the activity of Wnt/β‐catenin pathway.

### 3.5 DAB2IP inhibits β‐catenin nucleus accumulation through sequestration of RAC1

**FIGURE 5:**
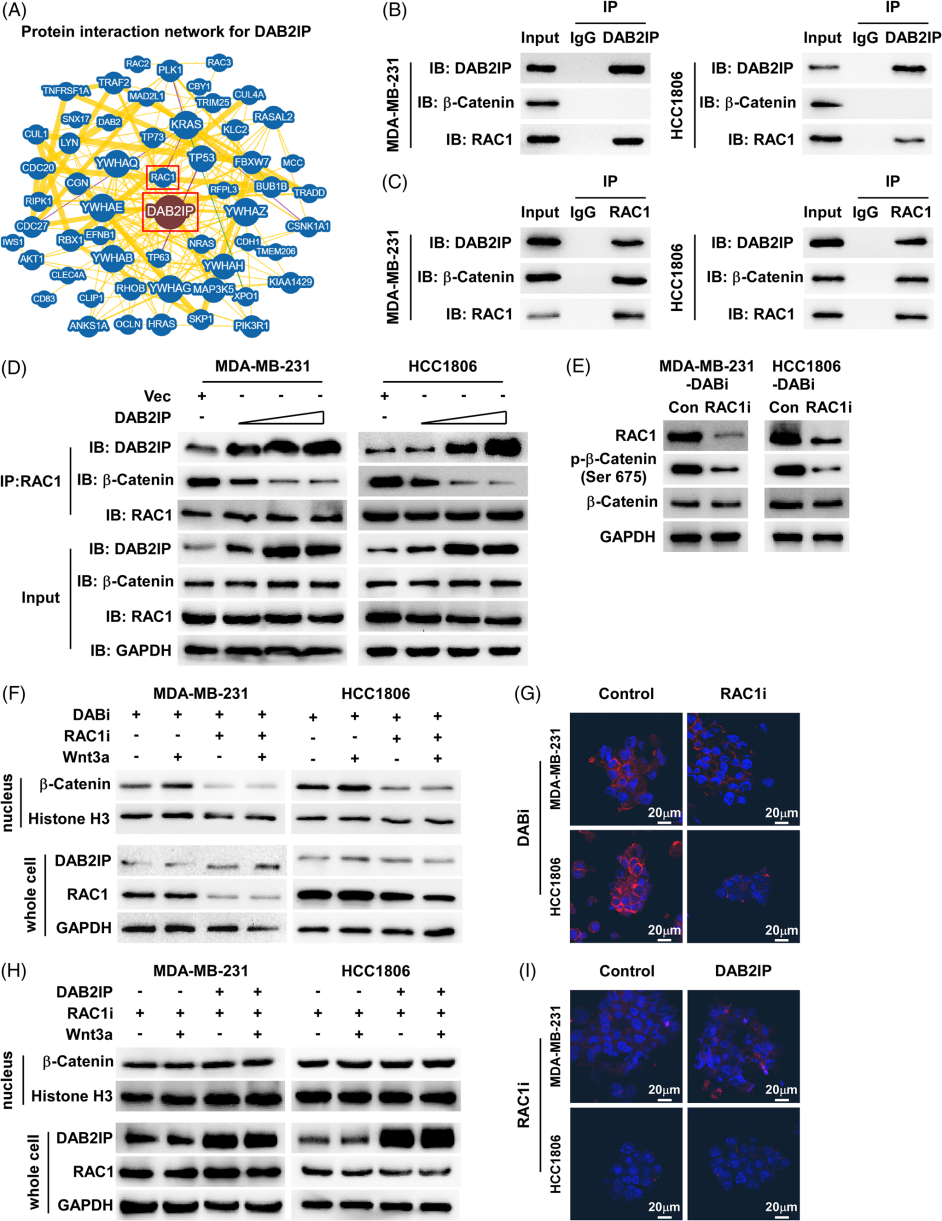
DAB2IP inhibits β‐catenin nucleus accumulation through sequestration of RAC1. (A) Protein interaction network of DAB2IP based on BioGRID database (https://thebiogrid.org/). (B and C) Co‐IP assay revealed the endogenous interaction between DAB2IP and RAC1 in triple‐negative breast cancer (TNBC) cells. (D) Competitive assay was conducted using Co‐IP assays: Co‐IP assays with endogenous RAC1 followed by Western blotting with DAB2IP and β‐catenin were conducted in triple‐negative breast cancer (TNBC) cells (5 × 10^5^) transfected with various concentration of DAB2IP (0, .5, 1, 2 μg). (E) Protein expression of phospho‐β‐catenin (Ser675) and β‐catenin in control and DAB2IP‐silencing (DABi#1) cells with Wnt3a stimulation (30 min). GAPDH was used as an internal control. (F) Protein expression of β‐catenin, DAB2IP and RAC1 in indicated cells with Wnt3a stimulation (30 min). Histone H3 was used as an internal control for nuclear protein, GAPDH was used as an internal control. (G) Representative images of β‐catenin (in red) stained using (immunofluorescence [IF]) in tumour spheroids formed by control and cells with RAC1‐inhibition. Tumour spheroids were stimulated by Wnt3a for 30 min. Cell nuclei were labelled with DAPI (in blue). Images were captured using confocal microscopy. Scale bar: 20 μm. (H) Protein expression of β‐catenin, DAB2IP and RAC1 in indicated cells with Wnt3a stimulation (30 min). Histone H3 was used as an internal control for nuclear protein; GAPDH was used as an internal control. (I) Representative images of β‐catenin (in red) stained using (IF) in tumour spheroids formed by control and cells with DAB2IP‐overexpression. Tumour spheroids were stimulated by Wnt3a for 30 min. Cell nuclei were labelled with DAPI (in blue). Images were captured using confocal microscopy. Scale bar: 20 μm

To further investigate the mechanism of how DAB2IP inhibits nucleus abundance of β‐catenin, we analyzed the protein interaction network of DAB2IP using the BioGRID database (https://thebiogrid.org/).^38^ Although the BioGRID database and Co‐IP (Co‐IP) assay did not show physical interaction between DAB2IP and β‐catenin, we discovered that RAC1 interacts with DAB2IP based on the BioGRID database and Co‐Immunoprecipitation (Co‐IP) (Figure 5A–C). RAC1 is a small guanosine triphosphate (GTP)‐binding protein, which was reported to induce β‐catenin nucleus import and increase protein stability through mediating β‐catenin phosphorylation (Ser675). The Co‐IP assay showed that both DAB2IP and β‐catenin are endogenously bound to RAC1 in TNBC cells (Figure 5B,C). Through exogenously expressing DAB2IP‐Flag and RAC1‐HA, we consistently observed the interaction between DAB2IP and RAC1 (Figure S5A,B). Thus, we hypothesized that DAB2IP might regulate the Wnt/β‐catenin signalling by targeting RAC1. As a previous study showed that DAB2IP is a GTPase‐activating protein (GAP), we first investigated whether DAB2IP inactivated RAC1 by inducing GTP hydrolysis. RAC1 Pull‐Down and detection assay indicated that DAB2IP did not affect the level of active GTP‐bound RAC1 (Figure S5C). Notably, the Co‐IP assay showed an increased level of DAB2IP‐RAC1 complex and a decreased level β‐catenin‐RAC1 complex in DAB2IP‐overexpression cells (Figure S5D). DAB2IP lowered the level of RAC1 binding to β‐catenin in a dose‐dependent manner (Figure 5D). RAC1‐inhibition reduced the expression of Ser675‐phospho‐β‐catenin in DAB2IP‐inhibition cells (Figure 5E). Western blotting and IF assay showed that RAC1‐inhibition prohibited β‐catenin nucleus accumulation in DAB2IP‐inhibition cells (Figure 5F,G). Tumour sphere assay indicated that the self‐renewal ability of cells with DAB2IP‐inhibition was weakened by RAC1‐inhibition (Figure S5E). RAC1‐inhibition significantly improved DOC response in cells with DAB2IP‐inhibition (Figure S5F). Also, DAB2IP‐overexpression did not further decrease β‐catenin nucleus accumulation in the presence of RAC1‐inhibition (Figure 5H,I). DAB2IP‐overexpression failed to attenuate CSC capacity in RAC1‐inhibition cells (Figure S5G,H). These data indicated that DAB2IP inhibited β‐catenin nucleus accumulation through competitive sequestration of RAC1.

### 3.6 DNA methylation associated with low expression of DAB2IP in TNBC

**FIGURE 6:**
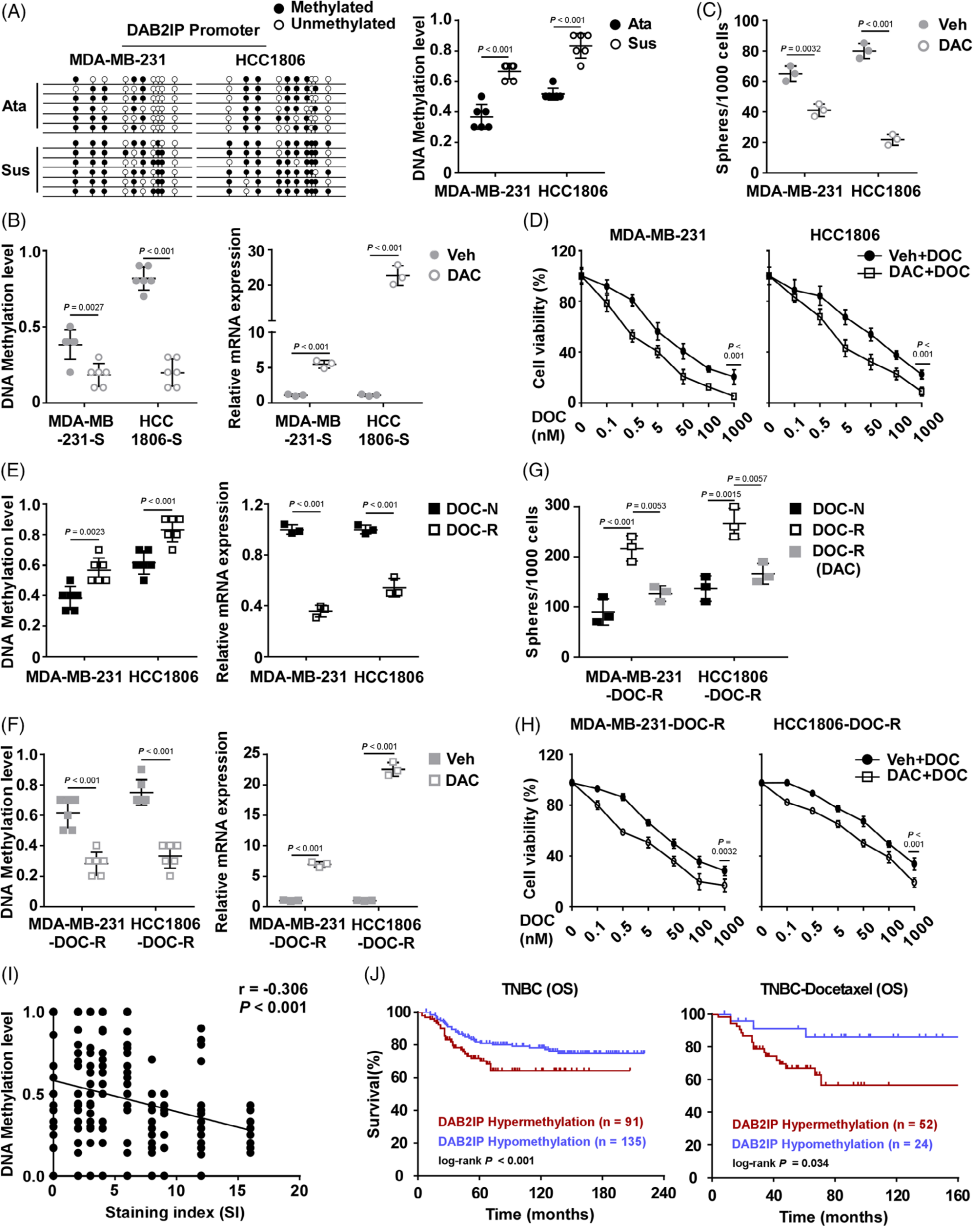
DNA methylation associated with low expression of DAB2IP in triple‐negative breast cancer (TNBC). (A) DNA methylation analysis (left panel) and quantification (right panel) of DAB2IP. (B) DNA methylation analysis (left panel) and mRNA expression of *DAB2IP* (*Transcript 1*) (right panel) in the suspension‐cultured spheroids with DAC treatment. (C) Quantification of tumour spheroids formed by the indicated TNBC cells. (D) Cell viability was assessed in cells with the indicated treatments. TNBC cells pretreated with a low dose of decitabine (DAC, 100 nM) or vehicle (veh) for 72 h were treated with the indicated concentrations of docetaxel (DOC, 72 h). (E) DNA methylation analysis (left panel) and quantitative real‐time polymerase chain reaction (qRT‐PCR) analysis (right panel) and of DAB2IP in the indicated cells. (F) DNA methylation analysis (left panel) and qRT‐PCR analysis (right panel) of DAB2IP in the DOC‐R cells with DAC treatment. (G) Quantification of tumour spheroids formed by the indicated TNBC cells. (H) Cell viability was assessed in cells with the indicated treatments. (I) Dot plot representing quantification of DAB2IP staining and DNA methylation levels in TNBC tissues (*n* = 226). (J) Kaplan–Meier curve of OS for TNBC patients (left panel) and TNBC patients with docetaxel‐based chemotherapy (right panel) using DAB2IP hypermethylation versus DAB2IP hypomethylation. In (A), (B), (C), (E) and (F), three replicates of each reaction are conducted, data are presented as the mean ± SD, and *p*‐values were determined by two‐tailed Student's *t* test. In (D) and (H), three replicates of each reaction are conducted, data are presented as the mean ± SD, and *p*‐values were determined by two‐way ANOVA. In (G), three replicates of each reaction are conducted, data are presented as the mean ± SD, and *p*‐values were determined by one‐way ANOVA. In (I), *p*‐values were determined by Pearson correlation coefficients. In (J), *p*‐values were determined by log‐rank test. ANOVA, analysis of variance; OS, overall survival.

Silencing of DAB2IP caused by promoter hypermethylation has been implicated in several cancers.^39^ We further investigated whether DNA methylation leads to DAB2IP low expression and confers aggressive tumour features in TNBC. We designed primers to evaluate DNA methylation status based on the CpG island distribution at the promoter region of DAB2IP (Figure S6A). We treated TNBC cells with Decitabine (DAC, 100 nM, 72 h), a DNA methylation inhibitor. Through detecting the level of DNA methylation and *DAB2IP* expression, we observed that TNBC cells (SUM159PT, HCC1806, MDA‐MB‐468 and MDA‐MB‐231) treated with DAC exhibited decreased level of *DAB2IP* DNA methylation (Figure S6B). DNA methylation inhibition increased DAB2IP mRNA expression (Figure S6C). These data indicated that DNA methylation associated with decreased DAB2IP expression in TNBC cells. To investigate the association between epigenetic silence of DAB2IP and tumour progression in TNBC, we detected the level of *DAB2IP* DNA methylation in CSC. MDA‐MB‐231 with high DAB2IP expression/low *DAB2IP* DNA methylation and HCC1806 with low DAB2IP expression/high *DAB2IP* were selected for suspension‐culture (Figure S6D). Compared to attachment‐cultured TNBC cells, cells with suspension culture exhibited an increased level of *DAB2IP* DNA methylation (Figure 6A). DAC treatment decreased the level of *DAB2IP* DNA methylation and restored DAB2IP mRNA expression in suspension‐cultured TNBC spheroids (Figure 6B). Inhibition of *DAB2IP* DNA methylation by DAC treatment significantly inhibited TNBC self‐renewal and increased the sensitivity to chemotherapy in TNBC cells (Figure 6C,D). Using Tumour‐sphere culture, our data showed that inhibition of self‐renewal in TNBC spheroids by DAC treatment could be attenuated by DAB2IP‐inhibition (Figure S6E). Cells with DAB2IP‐inhibition exhibited higher viability than control cells under DAC+DOC treatment (Figure S6F). These data indicated that the cancer inhibitory effect of DAC treatment required DAB2IP expression.

Next, we induced two DOC‐resistant (DOC‐R) cells (MDA‐MB‐231‐DOC‐R and HCC1806‐DOC‐R) (Figure S6G,H) to investigate whether DNA methylation of *DAB2IP* induces chemoresistance in TNBC cells. An increased DNA methylation level and decreased mRNA expression of *DAB2IP* were detected in DOC‐resistant cells (Figure 6E). DAC treatment restored mRNA expression of *DAB2IP* through inhibiting DNA methylation in DOC‐R cells (Figure 6F). Compared to chemotherapy‐naive (DOC‐N) cells, DOC‐R cells displayed enhanced self‐renewal capacity (Figure 6G). Consistently, the inhibition of DNA methylation inhibited CSC capacity and chemoresistance in DOC‐R cells (Figure 6G,H). At last, we detected the level of *DAB2IP* DNA methylation in TNBC specimens. Level of DNA methylation negatively correlated with DAB2IP protein expression level in TNBC tissues (Figure 6I). Survival analysis indicated that *DAB2IP*‐hypermethylation predicted with poor outcomes in TNBC patients and those with chemotherapy (including DOC‐based and non‐DOC‐based regimen) (Figure 6J and Figure S6I).

### 3.7 Decitabine inhibits DAB2IP methylation and improves the response to chemotherapy in TNBC

**FIGURE 7:**
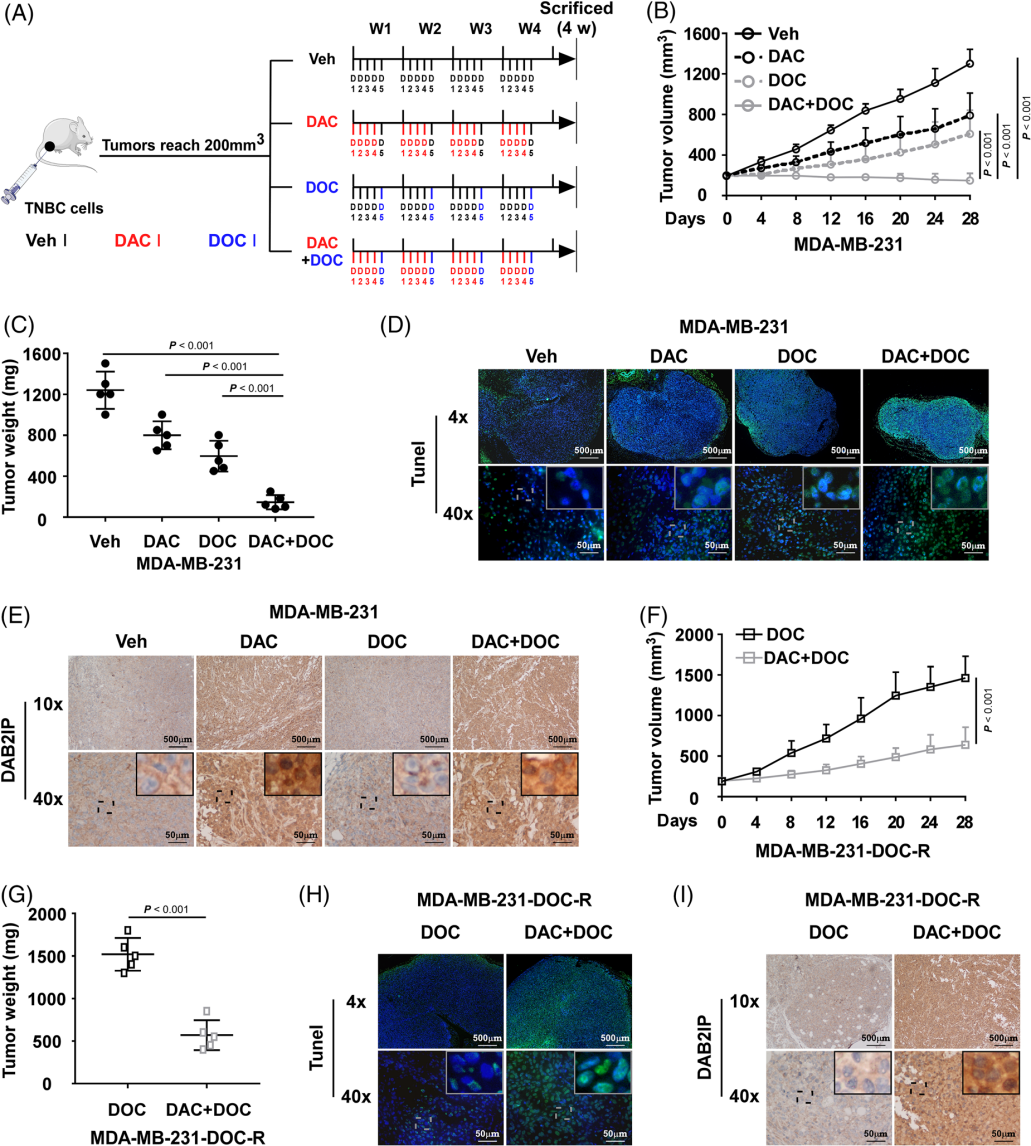
Decitabine inhibits DAB2IP methylation and improves the response to chemotherapy in TNBC. (A) Schematic diagram of the in vivo experimental strategies. MDA‐MB‐231 cells (1 × 10^6^) were subcutaneously injected into the right mammary fat pads of mice. Mice were randomly assigned to the veh group, DAC group, docetaxel (DOC) group or DAC+DOC group when tumours reached a volume of 200 mm^3^. Mice were sacrificed on day 28. (B) The volumes of tumours in the indicated groups (*n* = 5/group) were measured every 4 days and are shown by tumour growth curves. (C) Tumour weights of tumours in the indicated groups (*n* = 5/group). (D) Representative images of TUNEL staining of apoptotic cells in tumours treated with veh, DAC, DOC or DOC+DAC (*n* = 5/group). The cell nucleus was labelled with DAPI (in blue), and the apoptotic cell fraction was labelled with TUNEL (in green). Scale bars: 50 and 500 μm. (E) Representative images of DAB2IP staining using immunohistochemistry (IHC) in the indicated tumours. Scale bars: 50 and 500 μm. (F) The volumes of DOC‐R tumours with the indicated treatments (*n* = 5/group). (G) Tumour weights of DOC‐R tumours with the indicated treatments (*n* = 5/group). (H) Representative images of TUNEL staining of apoptotic cells in DOC‐R tumours treated with veh or DOC (*n* = 5/group). The cell nucleus was labelled with DAPI (in blue), and the apoptotic cell fraction was labelled with TUNEL (in green). Scale bars: 50 and 500 μm. (I) Representative images of DAB2IP staining using IHC in DOC‐R tumours with the indicated treatments. In (B) and (F), three replicates of each reaction are conducted, and data are presented as the mean ± SD, and *p*‐values were determined by two‐way ANOVA. In (C), three replicates of each reaction are conducted, data are presented as the mean ± SD, and *p*‐values were determined by one‐way ANOVA. In (G), three replicates of each reaction are conducted, data are presented as the mean ± SD, and *p*‐values were determined by two‐tailed Student's *t* test. ANOVA, analysis of variance, veh, vehicle.

As DAC restores DAB2IP expression in TNBC, whether DAC could be a therapeutic agent for chemoresistance in TNBC is worth further study. The animal assay was conducted to investigate the anti‐tumour effect of DAC (Figure 7A). Tumours treated with DAC before DOC grew much smaller and slower than the DOC group (Figure 7B,C and Figure S7A). The TUNEL assay showed that tumours of the DAC+DOC group exhibited a higher percentage of apoptotic cell than the DOC group (Figure 7D and Figure S7B). Tumours treated with DAC exhibited increased expression of DAB2IP (Figure 7E and Figure S7C). Moreover, DAC treatment improved chemotherapeutic response in chemoresistant tumours (Figure 7F–H and Figure S7D,E). DAC treatment increased DAB2IP expression in chemoresistant tumours (Figure 7I and Figure S7F). These data suggested that DAC treatment could be a potential treatment for chemoresistance in TNBC.

## 4 DISCUSSION

Although chemotherapy has markedly lowered the incidence of cancer recurrence in TNBC, 30% of cases still develop resistance to chemotherapy soon after chemotherapy.^40^ Finding the way to prohibit chemoresistance development and to treat chemoresistance are critical to improve cancer survival in TNBC. Since the up‐regulation of ABC transporter proteins and the hyper‐activation of ALDH, CSCs are associated with chemoresistance. With advantages of its potential for self‐renewal and drug resistance, CSCs would selectively survive the chemotherapeutic pressure and induces cancer relapse after chemotherapy. Investigation of the mechanism underlying CSC enrichment is critical to find valuable treatment for chemoresistance in TNBC. In our study, we observed significantly decreased DAB2IP expression in tumour spheroids and docetaxel‐resistant cells, indicating that low DAB2IP expression was associated with chemoresistance in TNBC. DAB2IP‐inhibition confers CSC capacity and chemoresistance through modulating Wnt/β‐catenin pathway in TNBC. DAB2IP inhibited β‐catenin nuclear accumulation through sequestration of RAC1. Our study revealed the role of DAB2IP in the modulation of chemoresistance in TNBC and might provide a promising therapeutic strategy.

The nuclear accumulation of β‐catenin is a sign for the activation of Wnt/β‐catenin pathway. In BC, β‐catenin transcriptionally activated target genes through binding LEF/TCF transcription factors in the cell nucleus, conferring CSC capacity, EMT, cancer metastasis and chemoresistance. Previous studies indicated that DAB2IP is involved in the control of Wnt/β‐catenin pathway in human cancers. In prostate cancer and glioblastoma multiforme, DAB2IP induced β‐catenin degradation by inhibiting GSK‐3β phosphorylation at S9. A recent study indicated that DAB2IP inhibited BC distant metastasis by inhibiting cancer EMT indicating that DAB2IP might also involve in the modulation of Wnt/β‐catenin pathway in BC. However, whether DAB2IP inhibited the activation of Wnt/β‐catenin pathway and the relevant mechanism remains unclear.

Notably, our study showed that DAB2IP neither induced β‐catenin Ser33/37 phosphorylation nor physically interacted with β‐catenin in TNBC. As loss of DAB2IP associated with nuclear abundance of β‐catenin, we further investigated the mechanism of how DAB2IP regulated Wnt/β‐catenin pathway in TNBC. Through protein interaction network and Co‐IP, we revealed that DAB2IP interacted with RAC1. RAC1 is a critical regulator for Wnt/β‐catenin signalling that it induced β‐catenin S675 phosphorylation through PAK1 and mediated β‐catenin nuclear transport.^14^ As a small GTP‐binding protein, RAC1 activity is regulated by GAP. However, our study showed that DAB2IP regulated RAC1‐mediated transport of β‐catenin without decreasing RAC1 activity. Co‐IP assays showed that DAB2IP decreased the interaction between RAC1 and β‐catenin, indicating that DAB2IP has a competitive association with RAC1 to inhibit β‐catenin nuclear transport. Thus, our study revealed DAB2IP/RAC1/β‐catenin axis as a novel regulatory mechanism for Wnt/β‐catenin pathway.

A previous study indicated that genome abnormity causing chemoresistance pre‐exist before chemotherapy, and these genomic aberrations commonly results from nongenetic mechanisms (including proteasome‐mediated degradation, transcriptional repression and epigenetic silencing).^6^ DNA methylation is an epigenetic process that binds a methyl group to CpG loci in the DNA sequence modulated by DNA methyltransferase (DNMTs), and it inhibits gene expression.^41^ Hypermethylation at the promoter region would lead to low expression of tumour suppressors, which renders tumour cells to survive chemotherapy. In BC, highly DNA methylation was widely discovered at promoters of cancer suppressors that modulate CSC ability, cell apoptosis and cell survival.^42^, ^43^ By decreasing DNMT expression, DAC inhibits genomic DNA methylation and induces re‐expression of a series of genes (including DAB2IP).^44^ Since DAB2IP is not the only gene regulated by DAC, DAC might act as a therapeutic agent for TNBC by affecting multiple pathways.^45^, ^46^ As we only investigated the effect of DAC on DAB2IP expression, further studies of genomic DNA methylation pattern variations caused by DAC treatment are required to comprehensively elucidate the anticancer effect of DAC in TNBC. Although we used a low dose of DAC, which is reported to be well tolerated, side effects and toxicity (including myelosuppression and asymptomatic increases in platelet counts without micronucleated erythrocytes) are inevitable.^47^, ^48^ The lack of biomarkers that predict sensitivity to DAC has prohibited the clinical application of DAC.^49^, ^50^ Our study revealed that low‐dose DAC treatment increased DAB2IP expression via inhibition of DNA methylation. Thus, the hypermethylation status of DAB2IP could be a biomarker to identify the population who would benefit from DAC treatment. Although clinical studies approving the above points of view are less, our study of the animal model indicated that the combination treatment (DAC‐pretreatment followed by DOC treatment) displayed a superior cancer‐inhibition effect over DOC treatment alone in TNBC. Whether the status of DAB2IP DNA methylation could act as a biomarker for potential benefit population of combination treatment (DAC+DOC) requires further studies.

## 5 CONCLUSIONS

**FIGURE 8:**
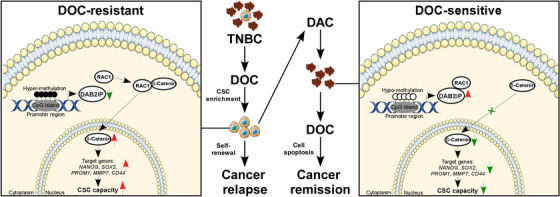
DAB2IP attenuates cancer stem cell (CSC) capacity and chemoresistance in triple‐negative breast cancer (TNBC). Hypermethylation at DAB2IP is associated with low DAB2IP expression and poor prognosis in TNBC. DAB2IP inhibited β‐catenin nuclear transport through competitive interaction with RAC1. Inhibition of Wnt/β‐catenin signalling by DAB2IP attenuates CSC capacity and chemoresistance in TNBC. Decitabine treatment re‐expresses DAB2IP through inhibiting DNA methylation and improves response to chemotherapy in TNBC.

In summary, we revealed that DAB2IP‐silence confers CSC capacity and chemoresistance in TNBC. DAB2IP inhibited β‐catenin nuclear transport through competitive interaction with RAC1. Hypermethylation at DAB2IP is associated with low DAB2IP expression and poor prognosis in TNBC. Decitabine treatment results in re‐expression of DAB2IP by inhibiting DNA methylation and could be a potential therapeutic strategy for chemoresistance in TNBC (Figure 8).

## CONFLICT OF INTEREST

All the authors declare that they have no conflicts of interest.

## Supporting information

Supporting Information

Supporting Information

Supporting Information

Supporting Information

Supporting Information

Supporting Information

Supporting Information

Supporting Information
