# Supplementary figures and images for: DAB2IP attenuates chemoresistance of triple‐negative breast cancer through sequestration of RAC1 to prevent β‐catenin nuclear accumulation
Source: Clin Transl Med. 2022 Dec 19;12(12):e1133. doi: 10.1002/ctm2.1133 (PMC9763535; doi:10.1002/ctm2.1133)

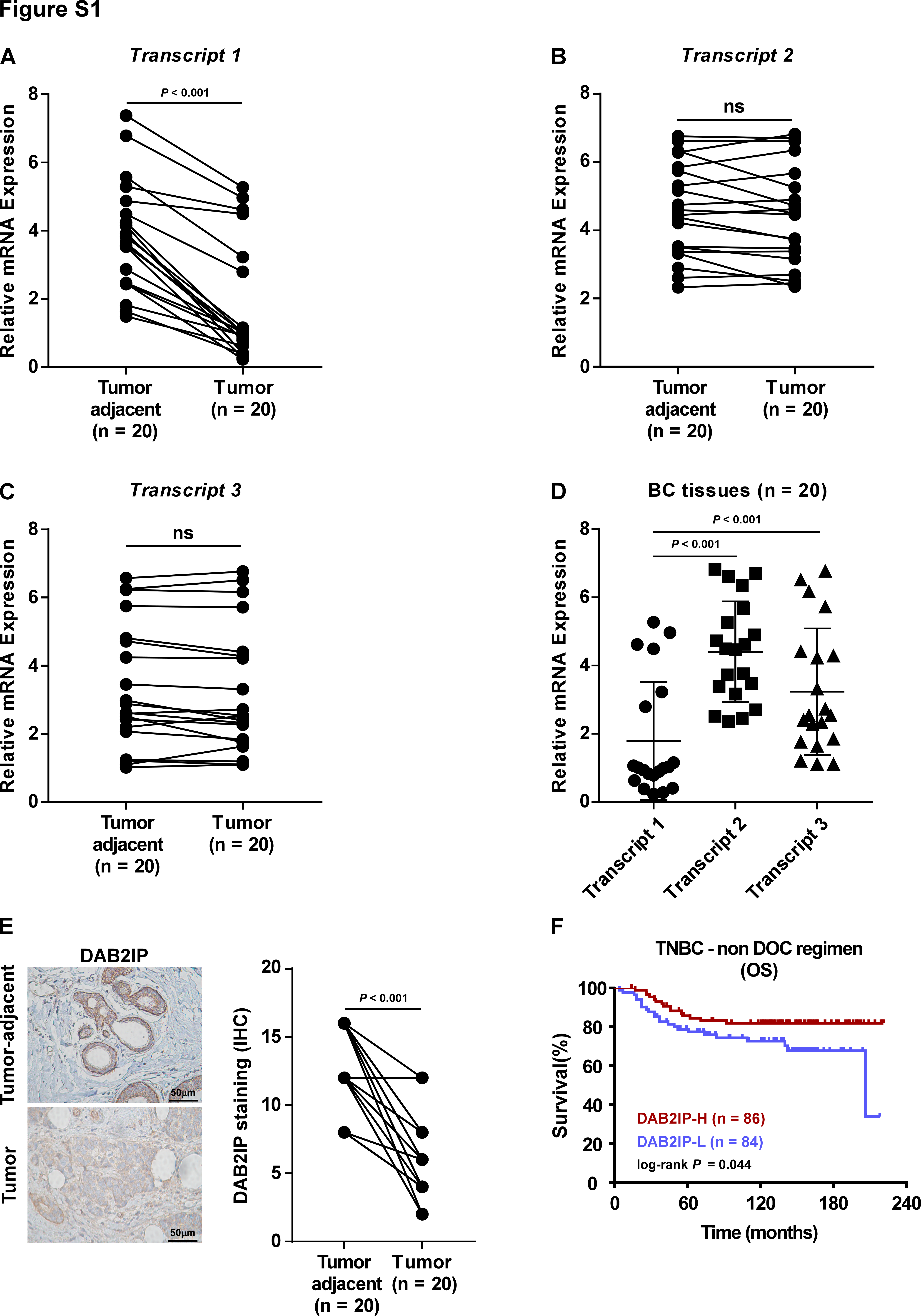

Supplement: Supplementary file 1 — Supporting Information [file CTM2-12-e1133-s002.tif]

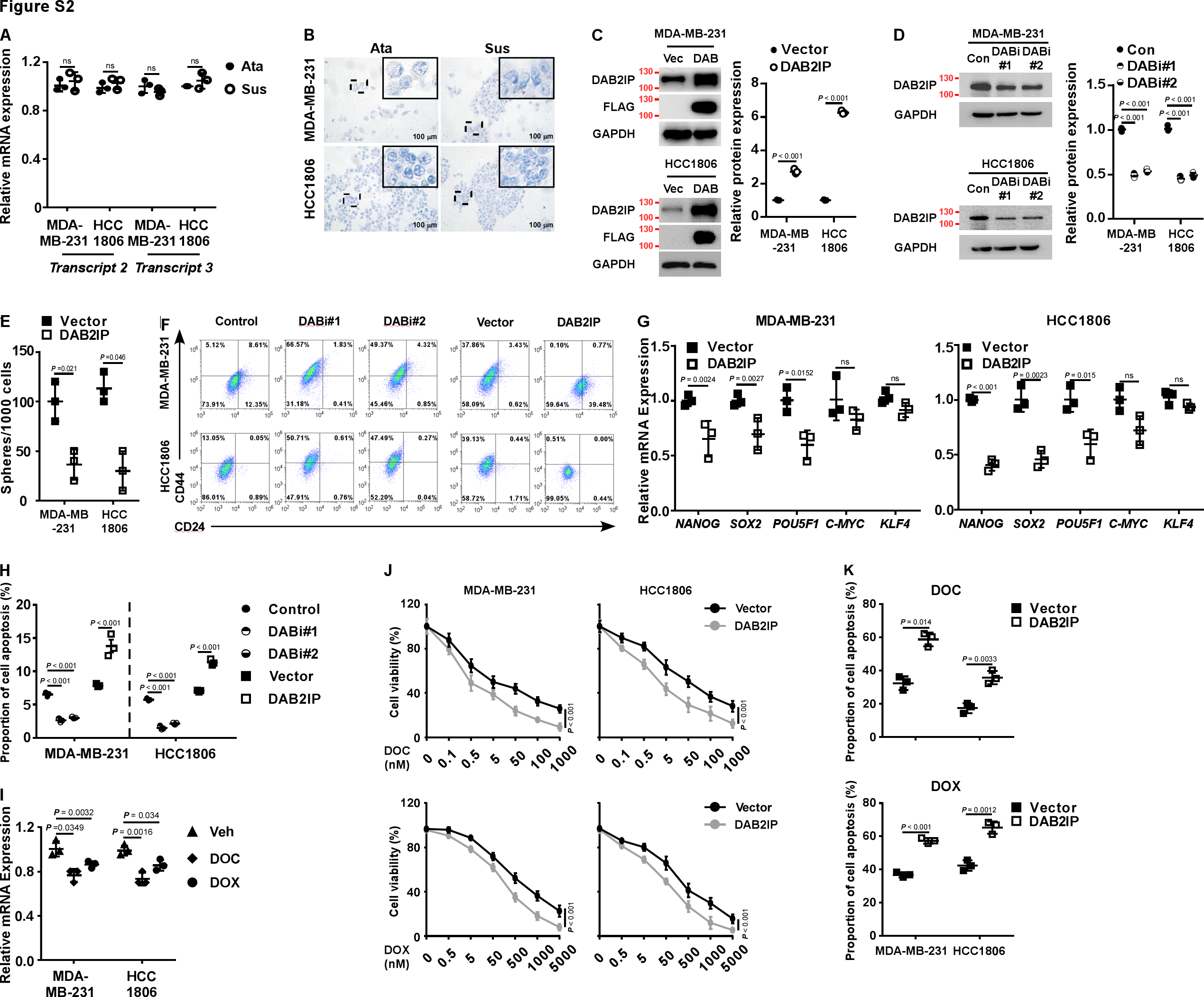

Supplement: Supplementary file 2 — Supporting Information [file CTM2-12-e1133-s001.tif]

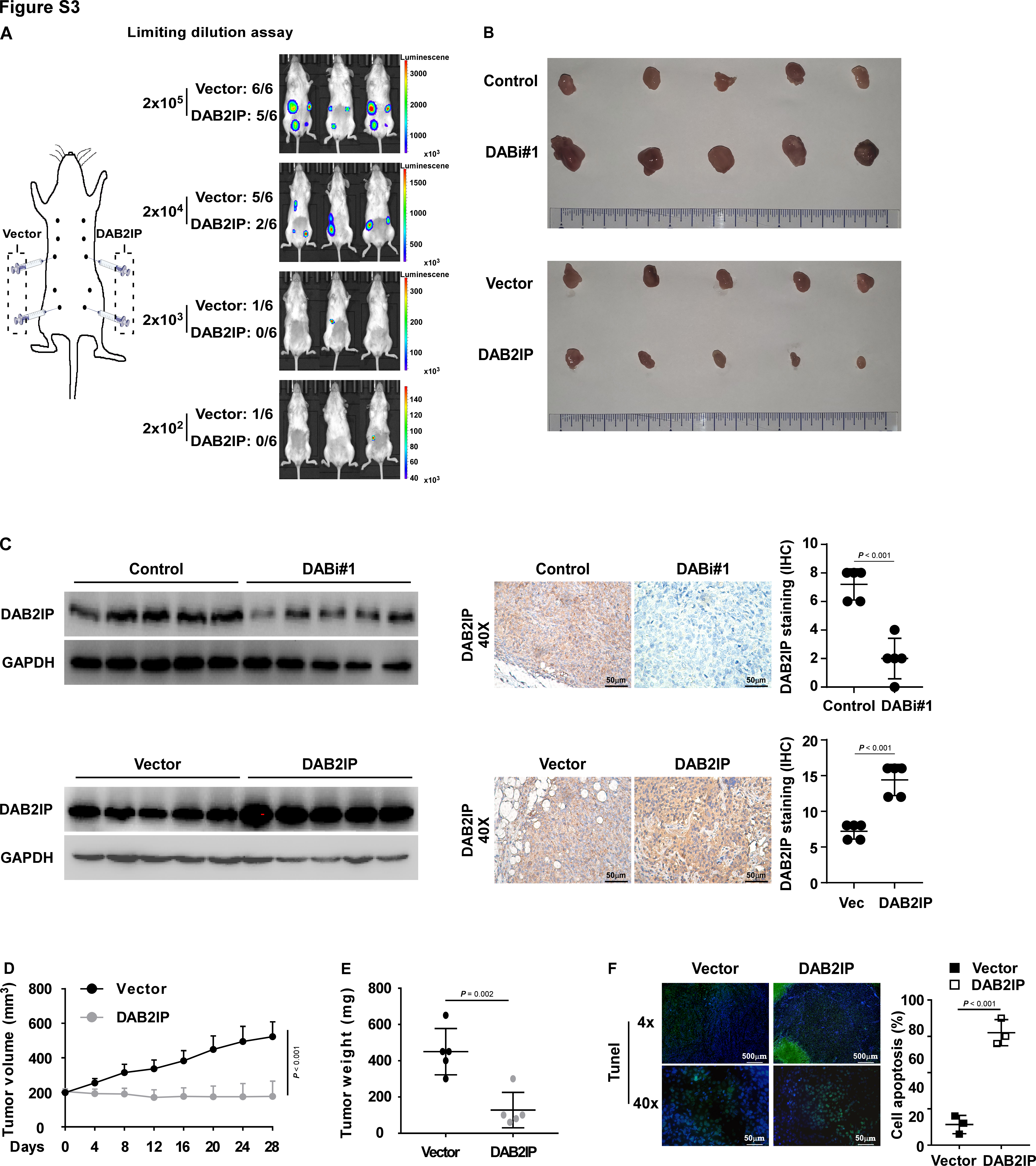

Supplement: Supplementary file 3 — Supporting Information [file CTM2-12-e1133-s003.tif]

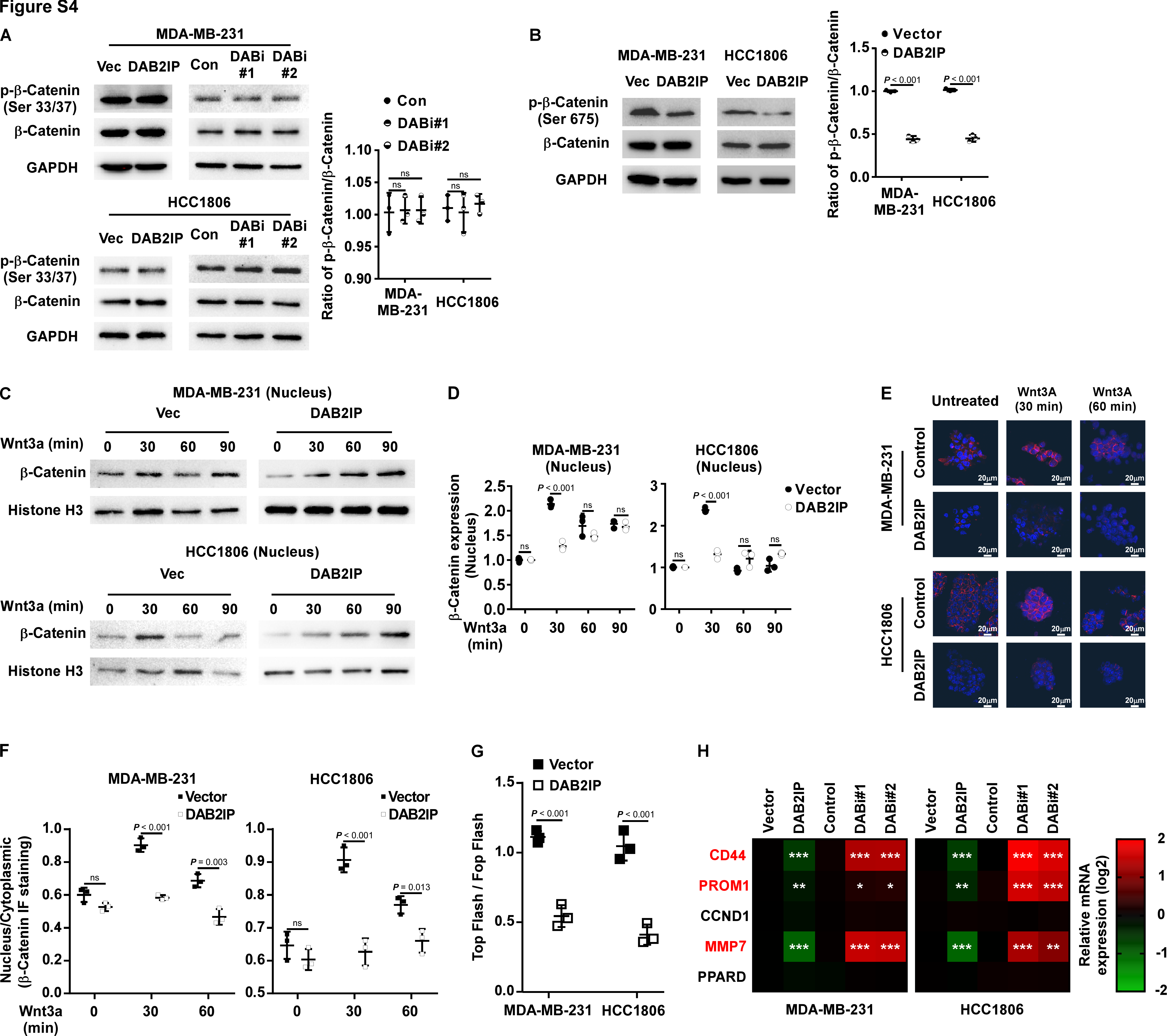

Supplement: Supplementary file 4 — Supporting Information [file CTM2-12-e1133-s007.tif]

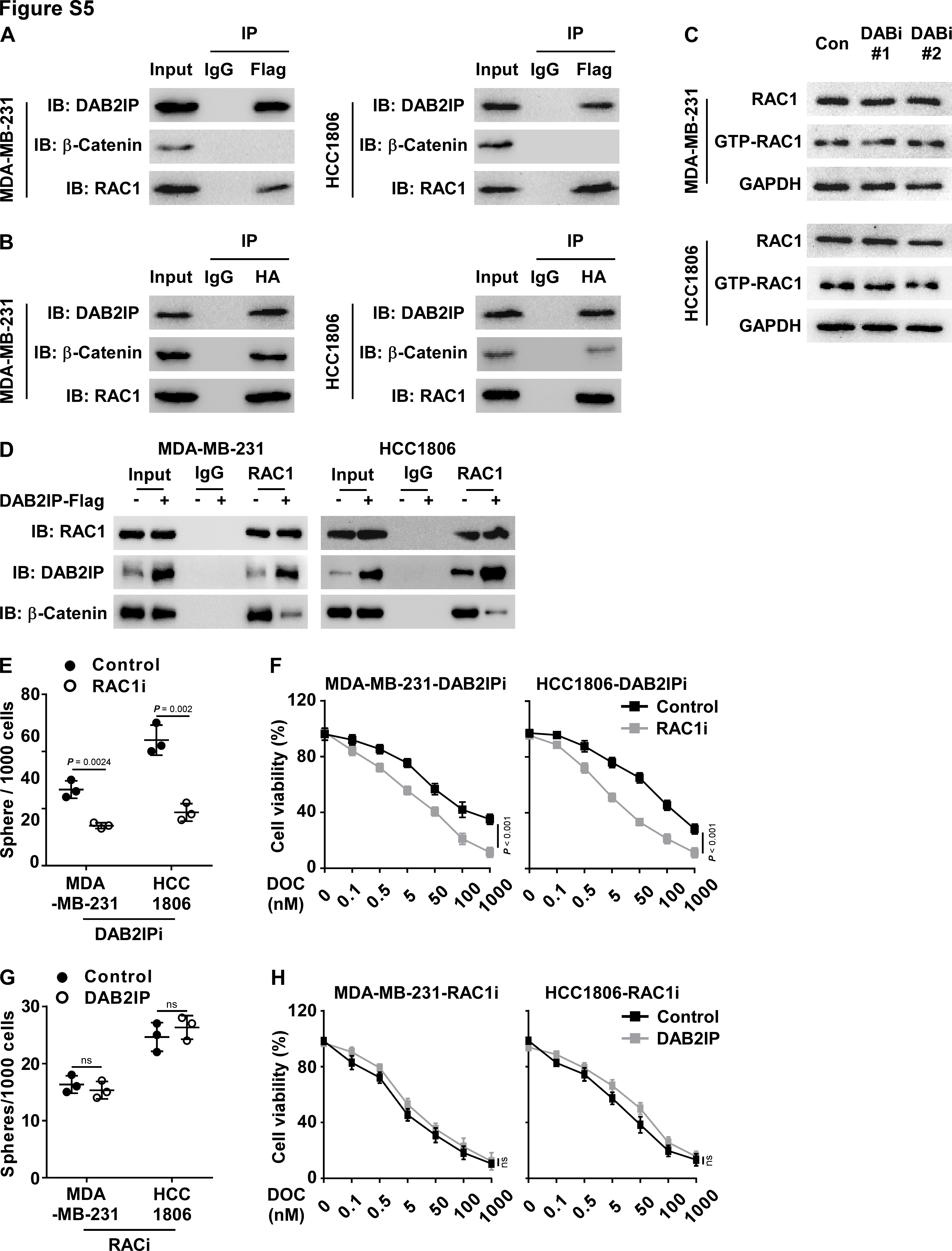

Supplement: Supplementary file 5 — Supporting Information [file CTM2-12-e1133-s008.tif]

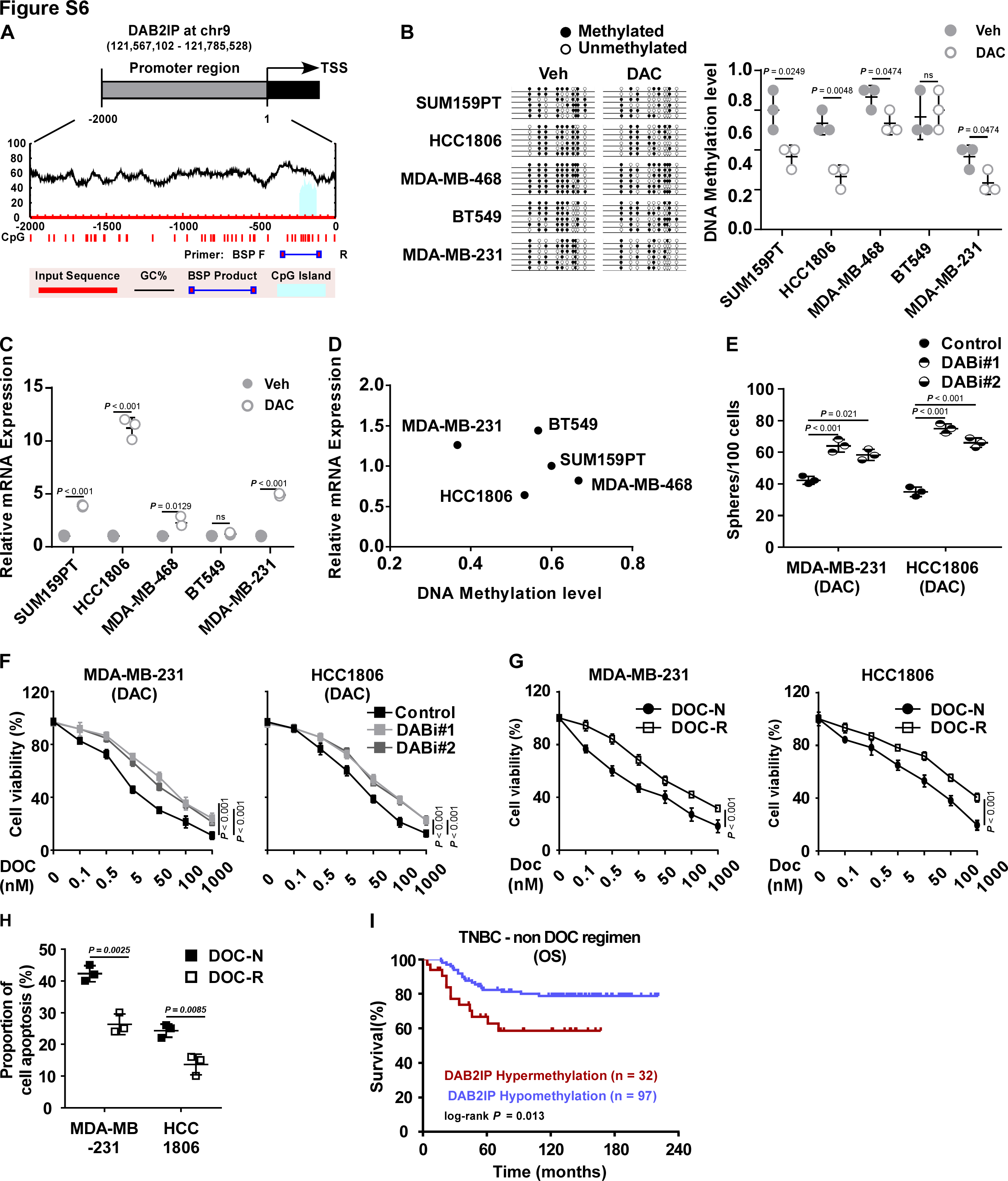

Supplement: Supplementary file 6 — Supporting Information [file CTM2-12-e1133-s004.tif]

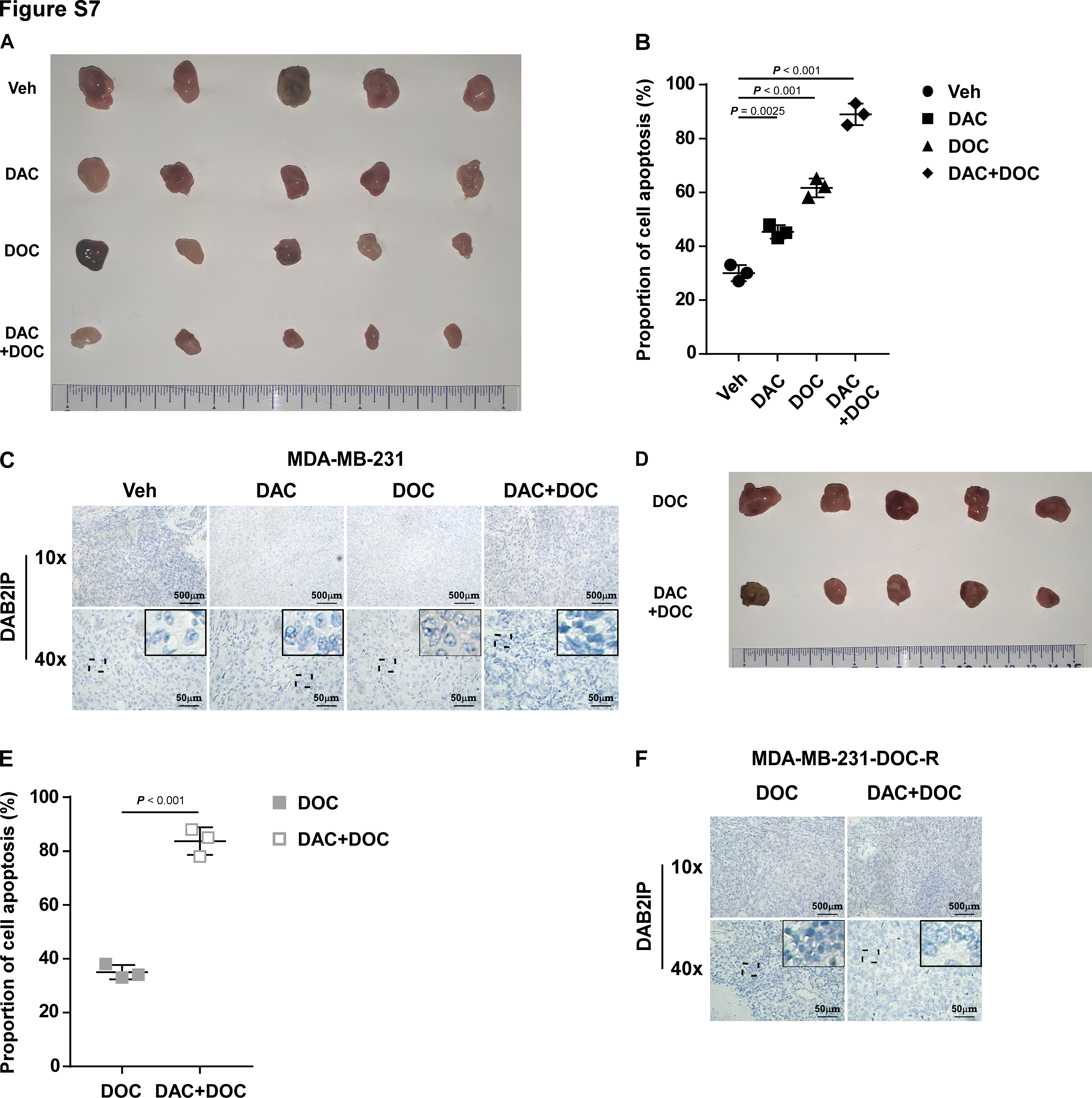

Supplement: Supplementary file 7 — Supporting Information [file CTM2-12-e1133-s005.tif]
