# Supplementary material for: DAB2IP attenuates chemoresistance of triple‐negative breast cancer through sequestration of RAC1 to prevent β‐catenin nuclear accumulation
Source: Clin Transl Med. 2022 Dec 19;12(12):e1133. doi: 10.1002/ctm2.1133 (PMC9763535; doi:10.1002/ctm2.1133)
Supplement: Supplementary file 8 — Supporting Information [file CTM2-12-e1133-s006.docx]

| **Supplemental Table 1. Clinicopathological characteristics of patients with TNBC** | | | | |
| --- | --- | --- | --- | --- |
| **Characteristics** |  |  |  | **No. of patients (%)** |
| **Age (y)** | | | | |
| **≤35** |  |  |  | 26 (8.5) |
| **35-50** |  |  |  | 145 (47.7) |
| **>50** |  |  |  | 133 (43.8) |
| **T classification** | | | | |
| **T0** |  |  |  | 2 (0.7) |
| **T1-2** |  |  |  | 264 (86.9) |
| **T3-4** |  |  |  | 32 (10.6) |
| **Unknown** |  |  |  | 6 (1.8) |
| **N classification** | | | | |
| **N0** |  |  |  | 165 (54.3) |
| **N1-2** |  |  |  | 98 (32.3) |
| **N3** |  |  |  | 38 (12.5) |
| **Unknown** |  |  |  | 3(0.9) |
| **Histological subtype** |  |  |  |  |
| **IDC** |  |  |  | 287 (94.4) |
| **ILC** |  |  |  | 2 (0.6) |
| **Other^*^** |  |  |  | 15 (5) |
| **HER2 status** |  |  |  |  |
| **0/1+ (by IHC)** |  |  |  | 259 (85.2) |
| **2+ (by IHC) and Negative status (by FISH)** |  |  |  | 45 (14.8) |
| **Menopause status** |  |  |  |  |
| **Premenopausal** |  |  |  | 186 (61.2) |
| **Menopausal** |  |  |  | 118 (38.8) |
| **Chemotherapy** |  |  |  |  |
| **Docetaxel-based regimen** |  |  |  | 104 (34.2) |
| **Other regimen** |  |  |  | 166 (54.6) |
| **Non-chemotherapy** |  |  |  | 30 (9.9) |
| **Unknown** |  |  |  | 4(1.3) |
| **Vital status (at follow-up)** | | | | |
| **Alive** |  |  |  | 223 (73.4) |
| **Dead** | |  |  | 81 (26.6) |
| **Relapse status** | | | | |
| **Relapse** |  |  |  | 86 (28.3) |
| **Relapse-free** |  |  |  | 218 (71.7) |
| **Expression of DAB2IP** | | | | |
| **Low expression** | |  |  | 184 (60.5) |
| **High expression** | |  |  | 120 (39.5) |
| **DNA methylation status** | |  |  |  |
| **Hyper-methylation** | |  |  | 91(29.9) |
| **Hypo-methylation** | |  |  | 135(44.4) |
| **Unknown** | |  |  | 78(25.7) |

**Abbreviation:** IHC, immunohistochemistry; FISH, fluorescent in situ hybridization; IDC, invasive ductal carcinoma; ILC, invasive lobular carcinoma.

**Other^*^:** IDC mixed with ILC or IDC mixed with other types of carcinomas (including: Invasive papillary carcinoma and medullary carcinoma).

**Supplemental Table 2. PCR primers in our study**

| **Gene** | **Forward primer (5'-3')** | **Reverse primer (5'-3')** |
| --- | --- | --- |
| ***DAB2IP (transcript 1)*** | CGCGCTACCAAACCATCAC | ATCATCAGGTCTGTCAGGAAGT |
| ***DAB2IP (transcript 2)*** | CAAACTGAAAGCCTGGTGGTG | GTCGTCACCTCGAAGCAGTA |
| ***DAB2IP (transcript 3)*** | GGTTCCTGAATGTGGTGTGTC | AAGTGCCAGGTCCCACATGA |
| ***NANOG*** | GGTCTCGTATTTGCTGCATCG | ACACTCGGTGAAATCAGGGT |
| ***SOX2*** | AACCAGCGCATGGACAGTTA | CGAGCTGGTCATGGAGTTGT |
| ***POU5F1*** | GGGTTGAGTAGTCCCTTCGC | TAGCCAGGTCCGAGGATCAA |
| ***C-MYC*** | CCCTCCACTCGGAAGGACTA | GCTGGTGCATTTTCGGTTGT |
| ***KLF4*** | ACCCTGGGTCTTGAGGAAGT | CAGCCCGAGCTACAAATCCC |
| ***CD44*** | CAGCTCATACCAGCCATCCA | TGGGGTGTGAGATTGGGTTG |
| ***PROM1*** | CACTACCAAGGACAAGGCGTTC | CAACGCCTCTTTGGTCTCCTTG |
| ***CCND1*** | TCTACACCGACAACTCCATCCG | TCTACACCGACAACTCCATCCG |
| ***MMP7*** | TCGGAGGAGATGCTCACTTCGA | GGATCAGAGGAATGTCCCATACC |
| ***PPARD*** | CGGACCTGGGGATTAATGGG | AGTATCACGTGCATGCCCAA |
| ***GAPDH*** | GACAGTCAGCCGCATCTTCT | GCGCCCAATACGACCAAATC |
| ***DAB2IP* (BSP assay)** | GGGTATTGGGGGTAAATAGAGTAGA | AAAACATTAAAAAAAACTAAAACAAC |

**Supplementary figure legends**

**Supplementary Figure S1. Low expression of DAB2IP predicted poor prognosis in TNBC.**

**(A)** The mRNA level of *DAB2IP* *Transcript 1* in paired tumor and tumor-adjacent noncancerous tissues.. **(B)** The mRNA level of *DAB2IP* *Transcript 2* in paired tumor and tumor-adjacent noncancerous tissues. **(C)** The mRNA level of *DAB2IP* transcript 3 in paired tumor and tumor-adjacent noncancerous tissues. **(D)** Comparison among three *transcripts* of *DAB2IP* in BC tissues. **(E)** DAB2IP expression in paired tumor and tumor-adjacent noncancerous tissues: (left panel) representative IHC image and (right panel) quantification of DAB2IP staining. **(F)** Kaplan–Meier curve of OS for TNBC patients with non-DOC-based chemotherapy using high expression of DAB2IP (DAB2IP-H) versus low expression of DAB2IP (DAB2IP-L).

In A-C, ***P***-values were determined by paired Student’s t test. In D, ***P***-values were determined by two-tailed Student’s t test. In E, ***P***-values were determined by two-way ANOVA. In F, ***P***-values were determined by log-rank test.

**Supplementary Figure S2. DAB2IP inhibits CSC capacities and chemoresistance in TNBC cells.**

**(A)** Comparison of the mRNA expression of *DAB2IP* (*Transcript 2* and *Transcript 3*) between attachment-cultured cells and suspension-cultured cells. GAPDH was used as an internal control. **(B)** Negative control for DAB2IP staining in TNBC cells or tumor-spheres. **(C)** Representative blots and quantification of DAB2IP protein expression in DAB2IP-overexpression TNBC cell lines. **(D)** Representative blots and quantification of DAB2IP protein expression in DAB2IP-inhibition TNBC cell lines. **(E)** Quantification of tumor spheres formed by the indicated cells. **(F)** Flow cytometry for CD44 and CD24 cell surface expression in the indicated cells. **(G)** qRT-PCR analysis of *NANOG*, *SOX2*, *POU5F1* and *C-MYC* in the indicated cells. **(H)** Cell apoptosis was evaluated by an Annexin V/PI assay using flow cytometry. The indicated cells were cultured under normal condition. **(I)** Comparison of the mRNA expression of *DAB2IP* (*Transcript 1*) between cells treated with DOC (5nM, 48h)/DOX (0.1μM, 48h) and cells treated with Veh. GAPDH was used as an internal control. **(J)** Cell viability was assessed in the indicated cells treated with DOC (upper panel) and DOX (lower panel). **(K)** Cell apoptosis was evaluated by an Annexin V/PI assay using flow cytometry. The indicated cells were treated with DOC (5nM, 48h; upper panel) and DOX (0.1μM, 48h; lower panel).

In A, C, E, G and K, three replicates of each sample are conducted and data are presented as the mean ± SD, ***P***-values were determined by two-tailed Student’s t test. In H, three replicates of each sample are conducted and data are presented as the mean ± SD, ***P***-values were determined by two-tailed Student’s t test and one-way ANOVA. In D and I, three replicates of each sample are conducted and data are presented as the mean ± SD, ***P***-values were determined by one-way ANOVA. In J, ***P***-values were determined by two-way ANOVA, ****P*** < 0.05, *****P*** < 0.01, ******P*** < 0.001.

**Supplementary Figure S3. DAB2IP inhibits CSC capacities and chemoresistance in TNBC cells.**

**(A)** The limiting dilution assay was used to evaluate tumor initiation capacity of the indicated cells. **(B)** Image of the indicated groups of tumors. **(C)** DAB2IP protein expression was assessed using Western blotting (left panel) and IHC (right panel) assay. **(D)** Volumes of tumors in the indicated groups (n = 5/group). **(E)** Tumor weights of tumors in the indicated groups. **(F)** Representative images (left panel) and quantification (right panel) of TUNEL staining of apoptotic cells in the indicated tumors treated with DOC (n = 5/group).

In C, ***P***-values were determined by two-tailed Student’s t test. In D, ***P***-values were determined by two-way ANOVA. In E and F, ***P***-values were determined by two-tailed Student’s t test.

**Supplementary Figure S4. DAB2IP inhibits Wnt/β-Catenin signaling.**

**(A)** Western blotting (left panel) of phospho-β-Catenin (Ser33/37) and β-Catenin and quantification (right panel) of phospho-β-Catenin (Ser33/37)/β-Catenin ratio in indicated cells with Wnt3a stimulation (30 min). **(B)** Western blotting (left panel) of phospho-β-Catenin (Ser675) and β-Catenin and quantification (right panel) of phospho-β-Catenin (Ser675)/β-Catenin ratio in indicated cells with Wnt3a stimulation (30 min). **(C-D)** Western blotting (C) and quantification (D) of nucleus β-Catenin in indicated TNBC cells stimulated with Wnt3a. Histone H3 was used as an internal control. **(E-F)** Representative images (E) and quantification (F) of β-Catenin (in red) staining in tumor spheres formed by the indicated cells. **(G)** Luciferase reporter assays of TOP Flash / Fop Flash reporters. **(H)** Heat map of relative mRNA expression of WNT/β-Catenin signaling downstream target genes in the indicated MDA-MB-231 and HCC1806 cells. The relative mRNA expression value is log2 transformed. Genes (*CD44*, *CCND1* and *MMP7*) which are significantly downregulated by DAB2IP-overexpression cell (Vector VS DAB2IP) and upregulated by DAB2IP-inhibition (Control VS DABi#1; Control VS DABi#2) are labeled in red.

In A, three replicates of each sample are conducted and data are presented as the mean ± SD, ***P***-values were determined by one-way ANOVA. In B, D, F and G, three replicates of each sample are conducted and data are presented as the mean ± SD, ***P***-values were determined by two-tailed Student’s t test. In H, three replicates of each sample are conducted, ***P***-values were determined by two-tailed Student’s t test and one-way ANOVA, ****P*** < 0.05, *****P*** < 0.01, ******P*** < 0.001.

**Supplementary Figure S5. DAB2IP inhibits β-Catenin nucleus accumulation through sequestration of RAC1.**

**(A, B)** DAB2IP-Flag and RAC1-HA were exogenously expressed in TNBC cells. Co-IP assay was used to reveal the exogenous interaction between DAB2IP and RAC1. **(C)** RAC1 activity was evaluated by Active Rac1 Pull-Down and detection assay. The protein level of GTP-bound RAC1 and RAC1 were detected by western blotting. **(D)** Competitive Co-IP assays with endogenous RAC1 followed by Western blotting with DAB2IP and β-Catenin were conducted in TNBC cells with DAB2IP-overexpression. **(E)** Effect of RAC1-inhibition on self-renewal ability in TNBC cells with DAB2IP-inhibition was assessed by tumor-sphere assay. **(F)** Effect of RAC1-inhibition on response to DOC in TNBC cells with DAB2IP-inhibition was assessed by cell viability assay. **(G)** Effect of DAB2IP-overexpression on self-renewal ability in TNBC cells with RAC1-inhibition was assessed by tumor-sphere assay. **(H)** Effect of DAB2IP-overexpression on response to DOC in TNBC cells with RAC1-inhibition was assessed by cell viability assay. Three replicates of each reaction are conducted and data are presented as the mean ± SD.

In E and G, three replicates of each reaction are conducted and data are presented as the mean ± SD, ***P***-values were determined by two-tailed Student’s t test. In F and H, three replicates of each reaction are conducted and data are presented as the mean ± SD, ***P***-values were determined by two-way ANOVA.

**Supplementary Figure S6. Low expression of DAB2IP was caused by DNA methylation in TNBC.**

**(A)** Primers for the bisulfite sequencing PCR was designed according to the location of CpG loci at the *DAB2IP* promoter region. **(B)** DNA methylation analysis (left panel) and quantification (right panel) in the indicated cells treated with DAC. **(C)** The qRT-PCR analysis of *DAB2IP* (*Transcript 1*) in cells with vehicle or DAC treatment. **(D)** Dot plot for relative *DAB2IP* mRNA (*Transcript 1*) expression (y axis) and *DAB2IP* DNA methylation level (x axis) in TNBC cells. **(E)** Quantification of tumor spheres formed by the indicated TNBC cells treated with DAC treatment. **(F)** Cell viability was assessed in cells with the indicated treatments. TNBC cells pretreated with a low dose of decitabine (DAC, 100 nM) or vehicle (veh) for 72 h were treated with the indicated concentrations of docetaxel (DOC, 72 h). **(G)** Cell viability was assessed in cells with the indicated treatments. **(H)** Cell apoptosis was evaluated by an Annexin V/PI assay using flow cytometry. The indicated cells were treated with DOC. **(I)** Kaplan–Meier curve of OS for TNBC patients with non-DOC-based chemotherapy using *DAB2IP* Hypermethylation versus *DAB2IP* Hypomethylation.

In B, C and H, ***P***-values were determined by two-tailed Student’s t test. In E, ***P***-values were determined by one-way ANOVA. In F and G, ***P***-values were determined by two-way ANOVA. In I, ***P***-values were determined by log-rank test.

**Supplementary Figure S7. Inhibition of DAB2IP methylation improves chemo-response in TNBC.**

**(A)** Image of the indicated groups of tumors. **(B)** Quantification of TUNEL staining of apoptotic cells in indicated groups of tumors. **(C)** Negative control for DAB2IP staining in TNBC tumors. **(D)** Image of the indicated groups of tumors. **(E)** Quantification of TUNEL staining of apoptotic cells in indicated groups of tumors. **(F)** Quantification of TUNEL staining of apoptotic cells in indicated groups of tumors.

In B, ***P***-values were determined by one-way ANOVA. In E, ***P***-values were determined by two-tailed Student’s t test.
